# Analysis of a Cu‐Doped Metal–Organic Framework, MFM‐520(Zn_1‐x_Cu_x_), for NO_2_ Adsorption

**DOI:** 10.1002/advs.202305542

**Published:** 2023-11-14

**Authors:** Zi Wang, Alena M. Sheveleva, Jiangnan Li, Zhengyang Zhou, Sergei Sapchenko, George Whitehead, Mark R. Warren, David Collison, Junliang Sun, Martin Schröder, Eric J. L. McInnes, Sihai Yang, Floriana Tuna

**Affiliations:** ^1^ Department of Chemistry University of Manchester Manchester M13 9PL UK; ^2^ Photon Science Institute University of Manchester Manchester M13 9PL UK; ^3^ State Key Laboratory of High Performance Ceramics and Superfine Microstructure, Shanghai Institute of Ceramics Chinese Academy of Sciences Shanghai 200050 China; ^4^ Diamond Light Source Harwell Science Campus Oxfordshire OX11 0DE UK; ^5^ College of Chemistry and Molecular Engineering, Beijing National Laboratory for Molecular Sciences Peking University Beijing 100871 China

**Keywords:** ENDOR, EPR spectroscopy, incommensurate modulation, MOFs, NO_2_ adsorption

## Abstract

MFM‐520(Zn) confines dimers of NO_2_ with a high adsorption of 4.52 mmol g^−1^ at 1 bar at 298 K. The synthesis and the incommensurate structure of Cu‐doped MFM‐520(Zn) are reported. The introduction of paramagnetic Cu^2+^ sites allows investigation of the electronic and geometric structure of metal site by in situ electron paramagnetic resonance (EPR) spectroscopy upon adsorption of NO_2_. By combining continuous wave and electron‐nuclear double resonance spectroscopy, an unusual reverse Berry distorted coordination geometry of the Cu^2+^ centers is observed. Interestingly, Cu‐doped MFM‐520(Zn_0.95_Cu_0.05_) shows enhanced adsorption of NO_2_ of 5.02 mmol g^−1^ at 1 bar at 298 K. Whereas MFM‐520(Zn) confines adsorbed NO_2_ as N_2_O_4_, the presence of monomeric NO_2_ at low temperature suggests that doping with Cu^2+^ centers into the framework plays an important role in tuning the dimerization of NO_2_ molecules in the pore via the formation of specific host‐guest interactions.

## Graphical abstract

The Cu‐doped MFM‐520(Zn_1‐x_Cu_x_) (x = 0.005, 0.01, 0.05), a mixed‐metal metal–organic framework, exhibits an intriguing reverse Berry distorted coordination geometry at the Cu^2+^ centers. This unique coordination structure is thoroughly examined using continuous wave and advanced pulsed EPR techniques. Additionally, this Cu‐doped framework demonstrates a remarkable 10% enhancement in NO_2_ capacity compared to its undoped counterpart.

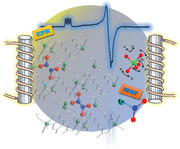

## 1 Introduction

Air pollution by nitrogen dioxide (NO_2_) is associated with serious environmental problems and health risks.^[^ ^1^ ^]^ Conventional porous materials based upon zeolites and activated carbons generally suffer from low uptake and/or severe structural degradation on adsorption of NO_2_ owing to the highly corrosive nature of this substrate.^[^ ^2^ ^]^ Recently, robust metal‐organic framework (MOF) materials have been confirmed to act as efficient and regenerable sorbents for NO_2_.^[^ ^3^ ^]^ The ultra‐microporous MOF, MFM‐520(Zn), [Zn_2_(L)]_∞_ (H_4_L = 4,4′‐bipyridine‐2,2′,6,6′‐tetracarboxylic acid), shows a very high NO_2_ uptake of 4.2 mmol g^−1^ at 298 K and low pressure (0.01 bar).^[^ ^4^ ^]^ In situ synchrotron X‐ray single crystal diffraction revealed that the bowtie‐shaped pores of MFM‐520(Zn) have an optimal size (6.6 × 4.0 Å^2^) to confine all adsorbed NO_2_ as diamagnetic N_2_O_4_; the absence of paramagnetic NO_2_ in NO_2_‐saturated MFM‐520(Zn) was confirmed by in situ EPR spectroscopy.

Recent studies on the catalytic degradation of NO_2_ over porous MOFs suggest that the introduction of Cu sites into the catalysts can greatly promote the conversion via enhanced adsorption of NO_2_.^[^ ^5^, ^6^ ^]^ Introduction of Cu^2+^ (3*d* ^9^ configuration) into such systems also gives an excellent probe for EPR spectroscopy, allowing the elucidation of electronic and geometric structure via the *g*‐matrix and the hyperfine interaction of the unpaired electron with the Cu^2+^ sites with surrounding nuclei that have non‐zero nuclear spin.^[^ ^7^, ^8^ ^]^ Although continuous‐wave (CW) EPR has been widely used to characterize the metal sites in MOFs,^[^ ^9^, ^10^ ^]^ the broad spectral linewidths and the superhyperfine interactions with ligands remain largely unresolved. Pulsed EPR techniques,^[^ ^11^ ^]^ such as electron nuclear double resonance (ENDOR) and hyperfine sublevel correlation spectroscopy (HYSCORE), can overcome this limitation of CW EPR.^[^ ^12^ ^]^

The ionic radii of Cu^2+^ (0.65 Å, 5‐coordinate) and Zn^2+^ (0.68 Å, 5‐coordinate) are similar,^[^ ^13^ ^]^ and thus partial replacement of Zn^2+^ centers in MFM‐520(Zn) by Cu^2+^ at low concentration is a viable route to afford well‐isolated Cu^2+^ centers. Here, we describe the synthesis, crystal structure, and in situ EPR studies of a series of Cu^2+^‐doped MFM‐520(Cu_x_Z_1‐x_) (x = 0.005, 0.01, 0.05) materials. The unusual form of the CW EPR spectrum of MFM‐520(Zn_0.995_Cu_0.005_) can be rationalized by invoking an unusual reverse Berry distorted coordination geometry at Cu^2+^, consistent with the known geometry of Zn^2+^ in this framework. This is also confirmed by ENDOR measurements on ligand nuclei (^14^N, ^1^H). Notably, a 10% enhancement of NO_2_ adsorption upon inclusion of Cu^2+^ sites is observed compared with the parent MFM‐520(Zn) (5.02 and 4.52 mmol g^−1^, respectively, at 298 K and 1 bar). Modulation of the incommensurate structure is observed upon doping with Cu^2+^ ions as well as via the packing of NO_2_ molecules that show an elongated intermolecular O_2_ N···NO_2_ distance [1.65(5)−1.87(4) Å] compared with MFM‐520(Zn) [1.46(7) Å]. We also report an in situ CW and pulsed EPR study of MFM‐520(Zn_0.995_Cu_0.005_) as a function of NO_2_ adsorption focussing on Cu^2+^‐NO_2_ interactions. This provides a rigorous analysis of the geometry of the Cu^2+^ site and affords key insights into the role of the metal site on NO_2_ adsorption.

## 2 Results and Discussion

### 2.1 Synthesis, Characterization, and Structure Determination

Cu^2+^‐doped MFM‐520 materials {[Zn_2‐2x_Cu_2x_(L)]·4H_2_O}_∞_ (*x* = 0.005, 0.01, and 0.05) were synthesized via hydrothermal reactions of ZnCl_2_, CuCl_2_ and H_4_L at 130°C for 6 days. *X* = 0.05 (or 5% dilution) is the highest ratio of doping that can be achieved under these conditions; higher levels of doping yield unknown phases by powder X‐ray diffraction (PXRD). The mixed‐metal MOFs were isolated as yellow–green microcrystalline powders, and the presence of Cu^2+^ confirmed by solid state UV–vis spectra (Figure S3, Supporting Information). Scanning electron microscopy (SEM) confirms the block morphology of the materials with crystal size distribution of 2–10 µm, and energy dispersive X‐ray spectroscopy (EDS) analysis confirms the homogenous distribution of both Cu and Zn in all samples (Figure S6, Supporting Information). Analysis of the ratio of Zn/Cu by inductively coupled plasma optical emission (ICP‐OES) gives good agreement with the stoichiometries used in the synthetic procedures (Table S1, Supporting Information). The PXRD patterns of all mixed‐metal MOFs are consistent with that of the parent MFM‐520(Zn) material, confirming retention of the framework structure and the 4^4^6^6^ topology^[^ ^14^ ^]^ (Figure S1, Supporting Information). Attempts to introduce Cu^2+^ sites via post‐synthetic modification by immersing the pristine MFM‐520(Zn) in an aqueous solution of CuCl_2_ failed, instead leading to the formation of a new [Cu_2_(L)] material showing a layered structure, which will be reported elsewhere. The retention of vibrational features of MFM‐520(Zn) in the mixed‐metal MOFs is confirmed by infrared (IR) spectroscopy (Figure S2, Supporting Information). Thus, these results confirm the successful preparation of MFM‐520(Zn_1‐x_Cu_x_) (*x* = 0.005, 0.01, and 0.05) materials with homogenous dilution of Cu^2+^ sites within the framework.

The presence of micropores in these MOFs has been confirmed by N_2_ adsorption isotherms at 77 K showing the typical Type‐I profile (Figure S5, Supporting Information). The Brunauer–Emmett–Teller (BET) surface areas of MFM‐520(Zn_1‐x_Cu_x_) (*x* = 0.005, 0.01, and 0.05) complexes were calculated from the isotherms to be 307, 303, and 309 m^2^ g^−1^, respectively, similar to that of MFM‐520(Zn) (313 m^2^ g^−1^). The retention of microporosity rules out the presence of clusters of copper oxide within the pores. Deolvated MFM‐520(Zn) has been reported to have an incommensurately modulated structure, being aperiodic in 3D space but periodic in (3 + 2)D space owing to the distortion of [ZnO_4_N] moieties.^[^ ^15^ ^]^ Synchrotron X‐ray single‐crystal diffraction at 150 K confirms that MFM‐520(Zn_0.95_Cu_0.05_) crystallises in triclinic system with a 4^4^6^6^‐type open framework with different modulation vectors [0.1240(3) (a* + b*) + 0.5c*] compared to MFM‐520(Zn) (Figure S7 and Table S3, Supporting Information), derived by partial replacement of Zn^2+^ by Cu^2+^ and variations in the distortion of the [Zn_0.95_Cu_0.05_O_4_N] moieties. Bond distances of M─N1, M─O1, and M─O2 (M = Zn_0.95_Cu_0.05_) lie in ranges of 1.92–2.09, 1.88–2.02, and 2.11–2.30 Å, respectively, accompanied by modulation of the electron density around the metal center with the formal oxidation state ranging from 1.79(3) to 2.19(1), as determined by bond valence sum (BVS) calculations (Figure S9, Supporting Information).

### 2.2 Continuous Wave EPR Study

**Figure 1:**
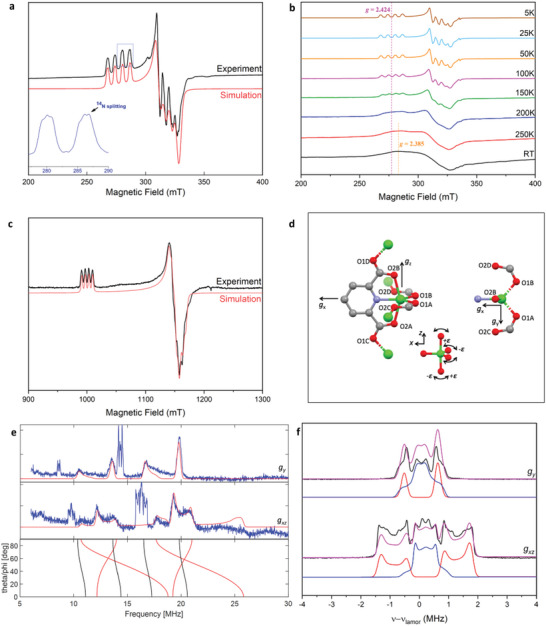
EPR spectra and views of MFM‐520(Zn_0.995_Cu_0.005_): a) X‐band CW spectrum at 5 K (black) and simulation (red) (inset: expansion of *g*_3_ feature, highlighting hyperfine to single ^14^N); b) X‐band CW EPR spectra from 5 K to room temperature; c) Q‐band CW spectrum at 5 K (black) and simulation (red); d) Zn site in the crystal structure of MFM‐520(Zn) (P4_2_2_1_2 space group) at 150 K along the orthogonal to (left), and down (right) the elongated O2A…Zn…O2B direction. Scheme: Zn (green), O (red), N (blue), C (grey). Dihedral angle between the ZnO1A/B and ZnO2A/B planes: 89.9(3)^o^. Distances (Å): Zn…N 2.009(6); Zn…O1A/B 1.957(4); Zn…O2A/B 2.210(2); Zn…O2C/D 2.883(7). Angles (^o^): O2A…Zn…O2B 152.4(3); O1A…Zn…O1B 99.1(3); N…Zn…O2A/B 76.2(1); N…Zn…O1A/B 130.4(3). The ***g*** (and ***A***) frame axes are labeled with the orientations fixed in the approximation of C_2v_ symmetry. Inset: schematic showing normal (+𝜺, towards SP) and reverse (−𝜺) Berry pseudo‐rotation pathways from trigonal bipyramidal (TBP) to square‐pyramidal (SP) geometry; e) Q‐band ENDOR spectra at 5 K, measured at B_0_ = 1004 (top) and 1142 mT (middle). Experimental data in blue; simulations in red (sharp peaks at *ca*. 15 MHz are overtones of ^1^H). Bottom: calculated ENDOR frequencies in the molecular xz plane at B_0_ = 1142 mT (red; 𝜃 = 0 (z) to 90^o^ (x) for 𝜑 = 0^o^) and xy plane at B_0_ = 1142 mT (black; 𝜑 = 0 (x) to 90^o^ (y) for 𝜃 = 90^o^); f) Q‐band (33.76 GHz) selective ENDOR experiment spectra (black). Simulation (H1: blue, H2: red, sum: magenta), including dipolar interaction and isotropic contributions to the hyperfine resulting from electron transfer from the metal into the σ system of the ligands.

CW EPR spectroscopy of MFM‐520(Zn_1‐x_Cu_x_) (x = 0.005, 0.01 and 0.05) shows broad bands at room temperature, with only partial resolution of the anisotropic *g*‐values (**Figure** **1**b). On cooling, the spectra sharpens and a shift of the low‐field (high *g*) features to lower field is observed. Below ca. 100 K, well‐resolved spectra are observed (Figure 1b) with resolution of the ^63,65^Cu hyperfine interaction (nuclear spin *I* = 3/2, combined 100% natural abundance). At X‐band, we observe hyperfine coupling to a single ^14^N nucleus (ca. 30 MHz; also observed in ENDOR), consistent with the Cu^2+^ dopant being incorporated into the Zn^2+^ site in rather than being “free” as solvated ions in the pores (Figure 1a). MFM‐520(Zn_1‐x_Cu_x_) (x = 0.005 and 0.05) give identical spectra with the linewidth increasing with increasing Cu^2+^ content due to long range Cu^2+^···Cu^2+^ interactions. Thus, the spectra confirm dilution of Cu^2+^ ions within the Zn^2+^‐based lattice (Figure 1c; Figure S13, Supporting Information).

The spectra at low temperature are near to axial, even at Q‐band resolution. Simulation of the spectra^[^ ^16^ ^]^ gives *g*_1,_ = 2.098, g_2,_ = 2.112, g_3_ = 2.424 with ^63^Cu hyperfine coupling constants |*A*_1_| = 140, |*A*_2_| = 125, |*A*_3_| = 206 MHz. These are unusual parameters for five‐coordinate Cu^2+^ complexes: they are very nearly axial despite the highly distorted geometry, *g*_3_ is very large, and |*A*_3_| is very small (with |*A*_1,2_| being unusually large). These observations can be rationalized in the context of the unusual coordination geometry at the metal site with C_2_ point symmetry based upon the averaged structure, but it approximates closely to C_2v_ (Figure 1d).^[^ ^14^ ^]^ The metal ion is bound by a pyridyl donor on the C_2_ axis and four carboxylates, two from the bound pyridyl (O2A/B), and two from the orthogonal layer (O1A/B). There are three short bonds to N1 and O1A/B [1.957(4)−2.009(6) Å] and two longer bonds to O2A/B [2.210(2) Å]. The latter also defines the largest angle at Zn [152.4(3)^o^] with the next largest angle being N···Zn···O1A/B [130.4(3)^o^].

The two parent geometries for five‐coordinate Cu^2+^ are elongated square‐pyramidal (SP) and compressed trigonal bipyramidal (TBP), with the former much more common.^[^ ^17^ ^]^ The distortion between these extremes is often quantified via the index τ_5_ = (𝛽−𝛼)/60^o^ where 𝛽 > 𝛼 are the two largest L···M···L angles.^[^ ^18^ ^]^ The relationship between EPR *g*‐values and TBP‐SP distortions is well understood: regular TBP (τ_5_ = 1) gives a $d_{z^{2}}$ ground state with characteristic *g*_x,y_ > *g*_z_ ≈ *g*_e_ (*g*_e_ is the free‐electron value, 2.0023), whilst regular SP (τ_5_ = 0) gives a $d_{x^{2}-y^{2}}$ ground state with characteristic *g*_z_ > *g*_x,y_ > *g*_e_ (defining z as the axial direction in either case). Intermediate geometries (0 < τ_5_ < 1) have rhombic *g*‐values due to the mixing of $d_{x^{2}-y^{2}}$ and $d_{z^{2}}$.^[^ ^19^ ^]^ This analysis assumes that the distortion lies on the conventional Berry pseudo‐rotation pathway between ideal TBP (D_3h_) and SP (C_4v_): this involves bending the two axial ligands (*z*, in D_3h_) away from one of the equatorial ligands (say, the *x*‐axis), whilst opening the angle between the other two, on a C_2v_ pathway (Figure 1d). Parameters for the metal site in MFM‐520 give τ_5_ = 0.37, indicating a significant distortion from either ideal geometry. This seems incompatible with the almost axial *g*‐values. However, the geometry lies on a *reverse* Berry distortion pathway (Figure 1d) with the two “axial” ligands (O2A/B) bent toward one of the equatorial ligands (N), and the angle between the other two equatorial ligands greatly decreased from 120^o^ (O1A···Zn···O1B 99^o^). This is a very rare geometry for Cu^2+^, and has only been observed in a few systems, for example [Cu(terpy)X_2_] (X = NCS^−^, Br^−^; terpy = 2,2′,6′,6′’‐terpyridine).^[^ ^20^, ^21^ ^]^ In these complexes, the central pyridyl defines the *C_2_* direction, and the distal pyridyls form the “axial” direction. This is partly due to the constraints imposed by the tridentate ligand, but also has a subtle variation with X. Other complexes [Cu(terpy)X_2_] form the more common distorted SP with an {N_3_X} basal plane.^[^ ^20^, ^21^ ^]^ The vast majority of small molecules of the [CuLX_2_] type (L = planar tridentate ligand), including those closely related to the metal site in MFM‐520, show distorted SP geometries with L defining the basal plane.^[^ ^22^ ^]^ In MFM‐520, the geometry at Cu^2+^ is constrained by the tridentate ligand, but the two additional ligands, carboxylates from pyridyls in the orthogonal layer in the lattice, are also constrained. Hence, the structure cannot deform to give the favored SP geometry.

The normal and reverse Berry distortion has important consequences for the electronic structure of Cu^2+^. In C_2v_ symmetry, the orientations of the ***g***‐matrix axes (but not their assignment to *g*_1_, *g*_2_, and *g*_3_, listed in numerical order) are fixed by symmetry, and the ***g*** and ***A*** matrices must be coincident. Choosing the labels in Figure 1d, the singly occupied molecular orbital (SOMO) can be described as a linear combination:^[^ ^23^ ^]^

(1) $$\left|SOMO\rangle \right.=\frac{a}{\sqrt{1+c^{2}}}\;\left(\left|z^{2}\rangle \right.+c\left|x^{2}-y^{2}\rangle \right.\right)$$

where *a* is the *3d* coefficient to the SOMO allowing for delocalization of spin density from the metal to the ligands. The coefficient *c* describes the symmetry‐allowed mixing of $d_{z^{2}}$ and $d_{x^{2}-y^{2}}$ that both transform as a_1_ in C_2v_. It should be noted that this can also be written in terms of a formal angle:^[^ ^19^ ^]^

(2) $$\left|SOMO\rangle \right.=a\left(\cos\beta \left|z^{2}\rangle \right.+\sin\beta \left|x^{2}-y^{2}\rangle \right.\right)$$

Equations (1) and (2) are equivalent with β  = tan ^−1^ *c* . With *c* = 0, the *3d* contribution to the SOMO is pure $d_{z^{2}}$ that corresponds to a TBP stereochemistry. A normal Berry rotation pathway gives a positive value for *c*, with the SP limit corresponding to *c* = +1/√3, giving the 3d contribution to the SOMO as $\frac{\sqrt{3}}{2}\left|z^{2}\rangle +\frac{1}{2}\right|x^{2}-y^{2}\rangle$. This can also be written as $d_{z^{2}-y^{2}}$, where *x* is the axial direction of the SP, and a reverse Berry rotation gives a negative value for *c*.

Perturbation theory gives the *g*‐values as:

(3) $$\Delta \;g_{x}=\frac{2a^{2}b_{yz}^{2}\lambda }{\Delta E_{yz}}\;{\left(\frac{c+\sqrt{3}}{\sqrt{1+c^{2}}}\right)}^{2}$$

(4) $$\Delta \;g_{y}=\frac{2a^{2}b_{xz}^{2}\lambda }{\Delta E_{xz}}\;{\left(\frac{c-\sqrt{3}}{\sqrt{1+c^{2}}}\right)}^{2}$$

(5) $$\Delta \;g_{z}=\frac{8a^{2}b_{xy}^{2}\lambda }{\Delta E_{xy}}\;\left(\frac{c^{2}}{1+c^{2}}\right)$$

where ∆*g*_i_ = *g*_i_ − *g*_e_, and ∆*E*_i_ is the energy gap from the ground state to the excited state, which is mixed in under spin‐orbit coupling in that orientation. 𝜆 is the spin‐orbit coupling constant for Cu^2+^ (830 cm^−1^, free ion^[^ ^24^ ^]^), and *b*_i_ accounts for delocalization in the excited state.

Equations (3)–(5) show that the near‐axial *g*‐values for MFM‐520(Zn_0.995_Cu_0.005_) with *g*_3_ >> *g*_1,2_ are only possible with |*c*| ≈ 1/√3. Given the “reverse” geometry, we must therefore be near the limit of *c* = −1/√3. This gives the SOMO as $\frac{\sqrt{3}}{2}|z^{2}\rangle -\frac{1}{2}\left|x^{2}-y^{2}\right\rangle$, which can also be written as $d_{z^{2}-x^{2}}$: in this limit the other a_1_ d‐orbital is $d_{y^{2}}$. Hence, the *g*‐values imply that we have a near pure $d_{z^{2}-x^{2}}$ ground state and that *g*_y_ (Figure 1d) is the unique and largest *g*‐value (*g*_3_). Unfortunately, we are unable to confirm this assignment by single crystal EPR because of the orthogonal layer structure in the MFM‐520 lattice, but such measurements have confirmed the large *g*‐value in the equivalent orientation (orthogonal to the terpy plane) in [Cu(terpy)(NCS)_2_] where *c* = −1/3.^[^ ^23^ ^]^ [Cu(terpy)(NCS)_2_] has larger X···Cu···X and L_ax_···Cu···L_ax_ angles [109.0(1) and 158.2(2)^o^ for X = Br, 98.1(3) and 158.9(2)° for X = NCS, respectively] compared to MFM‐520 [99.1(3) and 152.4(2)^o^, respectively], and has more rhombic *g*‐values (and a smaller *g*_y_). Thus, both the structural and EPR data are consistent with the Cu^2+^ site in MFM‐520 being “further along” the reverse Berry distortion than [Cu(terpy)(NCS)_2_]. It should be noted that a $d_{z^{2}-x^{2}}$ ground state is also consistent with the large ^14^N hyperfine coupling constant (*ca*. 30 MHz) to the pyridyl lying on the *x*‐axis (Figure 1d), and is similar to that observed in square planar [Cu(pyridine)_4_]^2+^.^[^ ^25^ ^]^

The ∆*E*_i_ terms in Equations (3)–(5) correspond to *d‐d* excitations. The solid‐state UV/vis/nIR spectrum of MFM‐520(Zn_0.995_Cu_0.005_) shows peaks at 960 and 580 nm (10.4 and 17.2 × 10^3^ cm^−1^, respectively; Figure S3, Supporting Information) that are not present in the parent Zn‐based material, and that we can assign to Cu^2+^ *d–d* transitions. Analysis of the reverse Berry distortion geometry gives the d‐orbital energies in the order $d_{z^{2}-x^{2}}\left(a_{1}\right)\gg d_{xz}\left(b_{2}\right)\approx d_{xy}\left(b_{1}\right)\approx d_{y^{2}}\left(a_{1}\right)>d_{yz}\left(a_{2}\right)$ for *c* = −1/√3, with the middle three separated by 2000 cm^−1^ (calculations performed on a model [CuCl_5_]^2−^ complex).^[^ ^22^ ^]^ The broad peak at 960 nm has a FWHM of *ca*. 3000 cm^−1^, and thus it is feasible that it consists of excitations to these multiple states. The narrow, weaker peak at higher energy can then be assigned to the *d*_yz_ excitation: this transition would be formally forbidden in C_2v_, but allowed in C_2_, which would also contribute to its weaker intensity. Substituting these excitation energies into Equations (3)–(5) with *c* = −1/√3, and taking *a* ^2^ = 0.8 (from analysis of the hyperfine interaction, see below) and *b* ^2^ = *a* ^2^ (i.e., assuming delocalization is similar for each d‐orbital), gives *g*_1_ = 2.06, *g*_2_ = 2.41, and *g*_3_ = 2.10. This is in remarkable close agreement with the experimentally observed values given the approximations in the theory.

On the basis of a near pure $d_{z^{2}-x^{2}}$ ground state, consistent with the *g*‐values, and approximating to axial symmetry [with *A*_⊥_ = (*A_x_* + *A_z_*)/2  and *g*_⊥_ = (*g_x_* + *g_z_*)/2 ; *A*_∥_ = *A_y_*  and *g*_∥_ = *g_y_* ], the following expressions can be derived for the Cu hyperfine interactions:

(6) $$A_{⊥}=P_{d}\;\left[-\kappa +\frac{2}{7}a^{2}+\frac{11}{14}\Delta g_{⊥}\right]$$

(7) $$A_{∥}=P_{d}\;\left[-\kappa -\frac{4}{7}a^{2}+\frac{6}{14}\Delta g_{⊥}+\Delta g_{∥}\right]$$

where *P*_d_ is the electron‐nuclear dipolar coupling parameter for ^63^Cu *3d* orbitals (+1197 MHz^[^ ^26^ ^]^). There are three contributions to the hyperfine constants in Equations (6) and (7): i) an isotropic Fermi contact term (proportional to 𝜅); ii) a spin‐dipolar contribution (proportional to *a* ^2^) relating to ground state d‐orbital electron spin density (see Equation (1)); and iii) orbital dipolar contributions via spin‐orbit coupling (proportional to ∆*g*). The orbital contribution is opposite in sign to the spin dipolar part and hence a large *g*‐shift, reflecting a low‐lying excited state (Equations (3)–(5)), will lead to a reduction in the observed hyperfine coupling. This latter effect, rather than extensive delocalization (small *a* ^2^) or significant $d_{z^{2}}$ mixing, is the cause of the unusually small hyperfine couplings observed in some other systems, e.g., in D_2d_ distorted [CuCl_4_]^2−^ and in ″type 0″ copper proteins.^[^ ^27^, ^28^ ^]^

Equations (6) and (7) can be combined to give:

(8) $$A_{∥}-A_{⊥}=P_{d}\;\left[-\frac{6}{7}a^{2}+\Delta g_{∥}-\frac{5}{14}\Delta g_{⊥}\right]$$

which removes uncertainty associated with the magnitude of 𝜅. Equation (8) can be solved for *a* ^2^ by substituting in the experimental *g* and *A* parameters. Assuming that the leading component of the Cu hyperfine coupling (*A_‖_*) is negative as predicted by theory for Cu, two solutions are possible. Taking *A_┴_* to be positive or negative gives *a* ^2^ = 0.77 or 0.52, respectively (i.e., ≈80% or 50% of the spin density is on the metal). The former is in keeping with the many simple Cu^2+^ complexes with O/N‐donor sets that have been analyzed by EPR spectroscopy, and thus this solution seems more likely. This would imply that the isotropic part of the hyperfine [*A_iso_* = (2*A*_⊥_ + *A*_∥_) /3] is rather small: this can be due to the large spin‐orbit contribution to *A*_iso_ [of the order *P_d_* (2Δ*g*_⊥_ + Δ*g*_∥_)/3] that is opposite in sign to the Fermi contact term,^[^ ^27^ ^]^ although valence shell spin polarisation effects can also be significant.^[^ ^29^ ^]^

In summary, the unusual form of the CW EPR spectrum of MFM‐520(Zn_0.995_Cu_0.005_) can be rationalized by an unusual reverse Berry distorted coordination geometry, and we can assign the observed *g*‐values with respect to the structure. The large *g* and small values for |*A*| are consistent with the weak ligand fields dominated by the four carboxylate donors, combined with the unusual geometry. Moreover, the data are consistent with the Cu^2+^ dopant adopting the parent structure of the [ZnNO_4_] moiety within the lattice, even though this would not be its preferred geometry. This is supported by the fact that we have not been able thus far to synthesize the neat Cu‐based analog MFM‐520(Cu). It should be noted that this study was limited to Cu doping level of 5% or lower. Higher levels of Cu^2+^ doping will distort the structure of the parent Zn^2+^ material and promote the formation of a 2D material through the direct combination of the H_4_L with Cu^2+^ with a more usual and expected distorted 6‐coordination at Cu^2+^. The observed reverse Berry distorted coordination geometry at Cu^2+^, which deviates from the preferred and usual geometry at Cu^2+^, can only be maintained at lower Cu^2+^ doping concentrations, where all the Cu^2+^ ions are confined within the parent framework structure defined by MFM‐520(Zn). We also note that the variable temperature linewidths and *g*‐values in the CW EPR spectra at higher temperature are indicative of dynamic effects involving the Cu^2+^ coordination sphere, which are then frozen out below *ca*. 100 K. Given the steric restrictions imposed by the lattice, it is highly unlikely that we are witnessing a dynamic average over two SP structures with O1A and O1B along the axes. However, distortions that open the O2A···Cu···O2B and/or O1A···Cu···O1B angles would be on the pathway toward normal Berry rotation and would result in a decrease in *g*_y_ and an increase in *g*_x_. Such a dynamic process at higher temperatures would be consistent with the observed broadened linewidths and the up‐field shift of the lowest‐field features in the EPR spectra. At higher loadings of Cu^2+^ (*x* = 0.05), there is a very broad signal of low intensity, which is more apparent in Q‐band spectra. This accounts for a small amount of the total signal intensity (<10%) (Figure S13, Supporting Information) and may be due to Cu^2+^ not incorporated into the lattice but in extra‐framework surface or pore sites and with higher mobility. Such species are often observed in EPR studies of Cu‐doped zeolites.^[^ ^30^ ^]^

### 2.3 ENDOR Spectra

In the CW X‐band EPR spectrum, there is partial resolution of ^14^N hyperfine (nuclear spin *I* = 1) on the *g*_∥_ feature that arises from the bound pyridyl (Figure 1e). To define this better, we have performed pulsed Davies ENDOR spectroscopy measurements. In ENDOR, neglecting quadrupole effects, peaks are observed at |*A*|/2 ± ν_n_ in the strong coupling regime when |*A*| > 2ν_n_, where *A* and ν_n_ are the hyperfine coupling and the nuclear Larmor frequency, respectively. A ^14^N splitting of *ca*. 30 MHz is observed in the CW EPR spectra suggesting that the ^14^N peaks (ν_N_ = 1.1 MHz at B_0_ = 350 mT) overlap with ^1^H peaks (ν_H_ = 14.9 MHz at B_0_ = 350 mT) at X‐band (Figure S27, Supporting Information), but are well‐resolved at Q‐band (ν_H_ and ν_N_ = 51 and 3.7 MHz, respectively, at B_0_ = 1200 mT). With the inclusion of ^14^N nuclear quadrupole coupling, each peak is further split by |3*P*| where *P* is the effective quadrupole coupling for a given orientation with respect to B_0_. Davies ENDOR spectra were measured on MFM‐520(Zn_0.995_Cu_0.005_) at the B_0_ positions indicated in Figure S18 (Supporting Information), corresponding to orientations along the molecular *y* axis (along “*g*_∥_”), and in the molecular *xz* (“*g*_⊥_”) plane with the axes as defined in Figure 1d. Along *g*_∥_ the simplest spectra are observed, with four well‐defined peaks centered at ca. 15 MHz (Figure 1e). In the g_┴_ plane, more complex spectra are observed as all orientations in the *xz* plane are being selected. Four of these almost coincide with those measured at *g*, implying a near axial set of ^14^N hyperfine and quadrupole parameters where the unique axis is within the *xz* plane. Because the ^14^N…Cu vector lies on the C_2_ axis with C_2v_ site symmetry, the ^14^N hyperfine and quadrupole matrices (***A*** ^N^ and ***P***, respectively) must have their principal axes coincident with each other and those of **g** and ***A*** ^Cu^. The data can be simulated with |*A*_x_^N^| = 38.0, |*A*_y_^N^| = 30.0 and |*A*_z_^N^| = 31.5 MHz, with a nuclear quadrupole coupling constant e^2^Qq/h = −4.4 MHz and asymmetry parameter 𝜂 = 0 corresponding to the quadrupole matrix principal values *P*_x_ = −2.2, *P*_x_ = 1.1 and *P*_z_ = 1.1 MHz (Figure 1e). The large component of the hyperfine is coincident with the large component of the quadrupole interaction, along *g*_x_, *i.e*., along the direction of the Cu···N bond (Figure 1e, bottom).

Quadrupole parameters are sensitive to the electric field gradient at the nucleus. NQR studies on pyridyl adducts with Lewis acids (LA) confirm the largest component to be along the direction of the lone pair of electrons at the ^14^N atom, i.e., along the N…LA axis.^[^ ^31^ ^]^ This has been confirmed in Cu…pyridyl complexes studied by orientation‐selective ENDOR,^[^ ^32^, ^33^, ^34^, ^35^, ^36^ ^]^ and is what we observe here for MFM‐520(Zn_0.995_Cu_0.005_). However, the |e^2^Qq/h| value here is larger than that typically observed in Cu···pyridyl complexes (typically −2.5 to −3 MHz; **Table** **1**).^[^ ^32^, ^33^, ^34^ ^]^ For example, planar [Cu(chelidamate)(dmf)] (dmf = N,N‐dimethlyformamide) is an interesting comparison to the Cu site in MFM‐520(Zn_0.995_Cu_0.005_) because chelidamate is a derivative of 2,6‐dipicolinate and has a value of e^2^Qq/h = −2.8 MHz.^[^ ^33^ ^]^ In the framework of the Townes‐Dailey model^[^ ^31^ ^]^ a smaller |e^2^Qq/h| implies a greater extent of electron donation from the ^14^N 𝜎‐donor (sp^2^ lone pair) orbital to the Lewis acid (LA), i.e., a stronger N…LA bond.^[^ ^30^ ^]^ Hence, the larger |e^2^Qq/h| value here would suggest a weaker Cu…N bond. A weaker N→Cu 𝜎‐donation could also be described as smaller Cu “hole”→N transfer, and would be expected to give rise to a smaller ^14^N hyperfine coupling. This is consistent with the ^14^N hyperfine values for MFM‐520(Zn_0.995_Cu_0.005_) that are smaller than for other related complexes (Table S6, Supporting Information).^[^ ^33^ ^]^ A crude calculation of the N 2p_x_ spin density can be obtained from:

(9) $$A_{∥}^{N}-A_{⊥}^{N}=\frac{6}{5}\;c^{2}P_{p}$$

where *c* ^2^ is the spin density, and *P*_p_ is the electron‐nuclear dipolar coupling parameter for N 2p orbitals (138.8 MHz).^[^ ^26^ ^]^ This gives *c* ^2^ = 4.4% for the Cu ion in MFM‐520(Zn_0.995_Cu_0.005_), lower than the 7.3% calculated from the hyperfine parameters for [Cu(chelidamte)(dmf)] which, as discussed above, is consistent with greater N2p→Cu donation in the latter. The isotropic part of the hyperfine is also smaller, but analysis of this in terms of valence s‐orbital density is more uncertain due to core polarisation effects. The small ^14^N hyperfine couplings and value of *c* ^2^ illustrate that the Cu─N bond in MFM‐520(Zn_0.995_Cu_0.005_) is relatively weak compared to other Cu─N coordinated molecules.

**Table 1:** Spin‐Hamiltonian parameter set extracted from Q‐band CW and ENDOR EPR spectra at 5 K.

MFM‐520(Zn_0.995_Cu_0.005_)	*g*_x,y,z_	*A* ^Cu^_x,y,z_/MHz	*A* ^N^_x,y,z_/MHz
Cu^2+^ (framework)	2.098, 2.424, 2.112	125, 206, 140	38.0, 30.0, 31.5
MFM‐520(Zn_0.95_Cu_0.05_)	*g*_x,y,z_	*A* ^Cu^_x,y,z_/MHz	*A* ^N^_x,y,z_/MHz
Cu^2+^ (framework)	2.089 2.111 2.424	120, 210, 140	38.0, 30.0, 31.5
Cu^2+^ (isotropic)	2.225	/	/
**NO_2_@ MFM‐520(Zn_0.995_Cu_0.005_)**	***g*_x,y,z_**	***A* ^Cu^_x,y,z_/MHz**	***A* ^N^_x,y,z_/MHz**
Cu^2+^ (framework)	A1: 2.101, 2.420, 2.101 A2: 2.095, 2.385, 2.095	140, 195, 140 140, 210, 140	38.5, 31.5, 33.2
NO_2_	2.004 1.9894 2.0007	/	144 130 185
**NO_2_@ MFM‐520(Zn_0.95_Cu_0.05_)**	***g*_x,y,z_**	***A* ^Cu^_x,y,z_/MHz**	***A* ^N^_x,y,z_/MHz**
Cu^2+^ (framework)	A1: 2.089, 2.417, 2.115 A2: 2.095, 2.376, 2.095	140 140 196 140 140 230	38.5, 31.5, 33.2
Cu^2+^ (broad signal)	2.190	/	/

Orientation selective ENDOR spectra centered at the proton Larmor frequency contain features of several doublets. Spectra collected at *g*_x,z_ are dominated by frequencies of *ca*. 4 MHz, while spectra at *g*_y_ are dominated by frequencies of *ca*. 2 MHz (Figure 1f). The ^1^H hyperfine matrix (*A*) at each proton atom includes contributions from an isotropic hyperfine interaction, *A^Hiso^*, and point dipole interactions (*A* ^dip^). We calculated the point‐dipole interactions (*A* ^dip^) for the nearest two sets of protons, the two protons of the pyridyl bound to the Cu (Cu···H1, ca. 4.9 Å), and those on the nearest pyridyl moieties in the orthogonal layer that bind to Cu via the carboxylates (Cu···H2, ca. 4.5 Å) (Figure S19 and Table S5, Supporting Information). Calculated spectra based on these parameters fail to reproduce the experimental spectra (Figure S20, Supporting Information). However, addition of an isotropic contribution of a_H1_ = 1.85 MHz and a_H2_ = 0.2 MHz gives good agreement (Figure 1f), consistent with a leaking of spin density into the bound pyridyl. The positive isotropic hyperfine coupling constant occurs when the metal and the ligand are bonded covalently, and this isotropic value is dominated by the unpaired electron transfer from the metal into the σ system of the ligands.^[^ ^32^ ^]^

### 2.4 Introduction of NO_2_ into MFM‐520(Zn_1‐x_Cu_x_)

**Figure 2:**
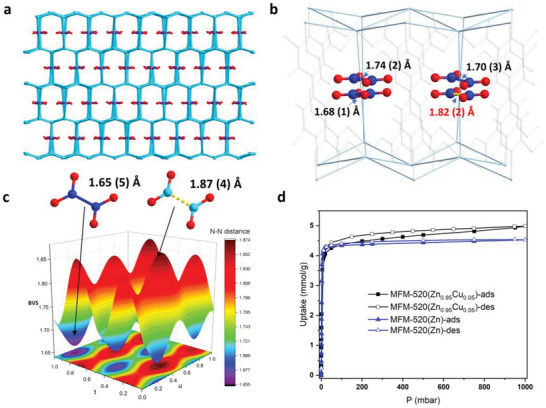
a) View of the crystal structures of NO_2_‐loaded MFM‐520(Zn_0.99_Cu_0.01_). [8 × 8 × 2] lattice indicates that the unit cell of the modulated structure is eight times that of the average structure along the *a* and *b* axes and twice along the *c* axis. b) Expanded view of (a), with different O_2_ N···NO_2_ distances highlighted; c) Maps of the bond valence sum (BVS) analyses of the N···N distance of N_2_O_4_ guest molecules as a function of the modulation vector *u* and *t* in NO_2_‐loaded MFM‐520(Zn_0.99_Cu_0.01_). The geometries of the N_2_O_4_ for the lowest and highest (highlighted in deep and light blue, respectively) BVS analysis maps are illustrated above the contour maps. Bond distances are in Å; d) NO_2_ adsorption isotherms of desolvated MFM‐520(Zn_0.95_Cu_0.05_) and MFM‐520(Zn) at 298 K.

We investigated NO_2_ adsorption in MFM‐520(Zn_1‐x_Cu_x_) via in situ synchrotron X‐ray single‐crystal diffraction. We have found that in NO_2_@MFM‐520(Zn) all NO_2_ molecules are in the form of the dimer N_2_O_4_, with an *N*–*N* distance of 1.46(7) Å.^[^ ^4^ ^]^ In contrast, the NO_2_ molecules trapped in MFM‐520(Zn_0.99_Cu_0.01_) show incommensurate modulation with *N*–*N* distances varying from 1.65(5) to 1.87(4) Å (**Figure** **2**a–c). Isothermal adsorption of NO_2_ in MFM‐520(Zn_0.95_Cu_0.05_) reveals a higher uptake of 5.02 mmol g^−1^ compared with that of MFM‐520(Zn) (4.52 mmol g^−1^) at 298 K and 1 bar, likely due to the increased entropic effects (Figure 2d).

**Figure 3:**
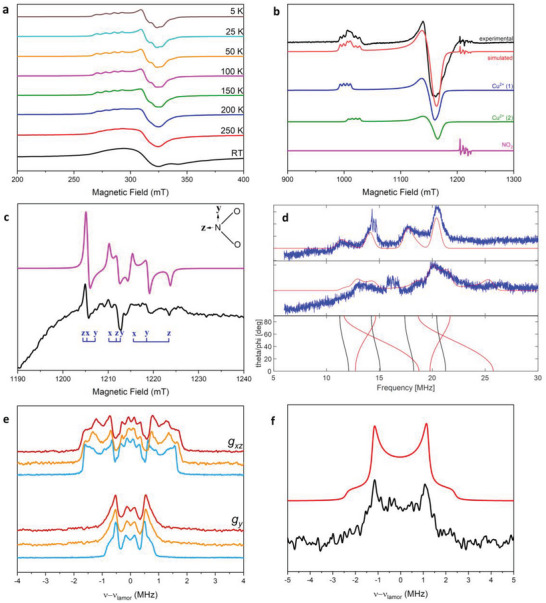
EPR spectra of NO_2_‐loaded MFM‐520(Zn_0.995_Cu_0.005_): a) X‐band CW EPR spectra from 5 K to room temperature; b) Q‐band CW spectrum at 5 K (black), and simulation (red). The simulation is the sum of the three different species Cu^2+^ (A1) (blue), Cu^2+^ (A2) (dark green), and NO_2_ (magenta); c) Expansion of NO_2_ signal from Figure 3b, experimental spectrum (black) and simulation (magenta); d) Q‐band ENDOR spectra at 5 K, measured at B_0_ = 1009 (top) and 1146 mT (middle). Experimental data in blue; simulations in red (sharp peaks at *ca*. 15 MHz overtones of ^1^H). Bottom: calculated ENDOR frequencies in the molecular *xz* plane at B_0_ = 1146 mT (red; 𝜃 = 0 (z) to 90^o^ (x) for 𝜑 = 0^o^) and *xy* plane at B_0_ = 1146 mT (black; 𝜑 = 0 (x) to 90^o^ (y) for 𝜃 = 90^o^); e) Q‐band (33.76 GHz) selective ENDOR experiment of prisitine (blue), NO_2_‐loaded MFM‐520(Zn_0.995_Cu_0.005_) (orange) and NO_2_‐loaded MFM‐520(Zn_0.95_Cu_0.05_) (deep red); f) ENDOR spectrum collected at the field position of NO_2_ signal (1198.3 mT) (black). The simulated spectra (red) show low sensitivity of polar angles between N‐H vector and *g*‐tensor frame, but the N‐H distance of 3.2 Å can be readily obtained from the simulation model.

In situ EPR spectroscopy was used to track the changes at Cu^2+^ before and after NO_2_ loading. On NO_2_ loading, similar variable temperature CW EPR spectra are observed (**Figure** **3**a), with narrowing of the spectra on cooling. At low temperatures, two distinct Cu^2+^ signals are observed (Figure 3b), both with similar parameters to those for the unloaded material. The *g*_∥_ regions overlap, but with sufficient resolution at Q‐band to define *g*_∥_ and *A*_∥_ (Table 1). These differences indicate slight changes of the Cu^2+^ environment, consistent with a change in structural modulation or direct interaction between Cu^2+^ and NO_2_ molecules. Furthermore, an additional weak signal of monomeric NO_2_ was observed at low temperature (Figure 3c), consistent with the N···N distances from X‐ray diffraction results (Figure 2b,c). The EPR parameters for the NO_2_ signal are comparable with those of adsorbed NO_2_ in other porous media (Table 1; Table S7, Supporting Information),^[^ ^3^ ^]^ suggesting that the introduction of Cu^2+^ centers into the framework plays an important role in tuning the dimerization of NO_2_ molecules via formation of specific modulated host‐guest interactions.

The introduction of NO_2_ also results to changes to ^1^H and ^14^N hyperfine, measured at the Cu^2+^ signal in the Davis ENDOR spectra (Figure 3d,e). The ^14^N hyperfine is slightly increased, suggesting a slightly stronger Cu···N bond upon loading of NO_2_; a consistent change was also observed for the quadrupole parameter that decreases from 4.4 to 4.0 upon loading of NO_2_ (Table S5, Supporting Information). Comparing the ^1^H signal before and after NO_2_ loading, we observe subtle changes in the shape of spectra, as well as a broadening in the *g*_y_ orientation. Such effects can result from the distribution of molecular conformations due to structural heterogeneities, commonly observed in biological systems.^[^ ^37^, ^38^ ^]^ This is very likely to occur in NO_2_‐loaded MFM‐520(Zn_0.995_Cu_0.005_) because of the structural heterogeneities from the incommensurate modulation of the structure. ENDOR spectra at the field position of the NO_2_ signal are consistent with nearest interactions with framework protons (based on a point dipole O_2_N···H interaction) of 3.2 Å, which is in excellent agreement with the N···H distance of 3.13‐3.41 Å from the X‐ray diffraction analysis (Figure 1f; Figure S8a, Supporting Information).

## 3 Conclusion

The geometry and local environment of Cu^2+^ in MFM‐520(Zn_0.995_Cu_0.005_) was probed by CW and pulsed EPR spectroscopy, and the unusual form of the CW EPR spectrum rationalized by proposing a reverse Berry distorted coordination geometry, the first such example in a periodic material. ^14^N and ^1^H hyperfine couplings observed in the pulsed ENDOR spectra further confirm the successful doping of Cu^2+^ into the Zn^2+^ framework sites with even distribution. Introduction of Cu^2+^ to MFM‐520(Zn) gives further structural incommensurate modulation and changes the degree of dimerization of adsorbed NO_2_ molecules. Enhanced adsorption of NO_2_ has been observed upon doping of Cu^2+^ centers in MFM‐520(Zn_1‐x_Cu_x_) materials from 4.52 to 5.02 mmol g^−1^ and loading of NO_2_ generates two Cu^2+^ signals with slightly different spin‐Hamilton parameters confirming changes of electron distribution of Cu^2+^ sites on loading of NO_2_. Monomeric NO_2_ molecules are observed spectroscopically in NO_2_‐loaded MFM‐520(Cu_0.005_Zn_0.995_) even at low temperature (*T* = 5 K), in contrast to the parent MFM‐520(Zn) material.

[CCDC 2259399 and 2259252 contain the supplementary crystallographic data for this paper. These data can be obtained free of charge from The Cambridge Crystallographic Data Centre via www.ccdc.cam.ac.uk/data_request/cif.]

## Conflict of Interest

The authors declare no conflict of interest.

## Supporting information

Supporting Information

Supporting Information

## Data Availability

The data that support the findings of this study are available from the corresponding author upon reasonable request.
