# Supplementary material for: Analysis of a Cu‐Doped Metal–Organic Framework, MFM‐520(Zn1‐xCux), for NO2 Adsorption
Source: Adv Sci (Weinh). 2023 Nov 14;11(1):2305542. doi: 10.1002/advs.202305542 (PMC10767414; doi:10.1002/advs.202305542)
Supplement: Supplementary file 1 — Supporting Information [file ADVS-11-2305542-s001.pdf]

ADVANCED SCIENCE
Open Access

Supporting Information

for *Adv. Sci.*, DOI 10.1002/advs.202305542

Analysis of a Cu-Doped Metal–Organic Framework, MFM-520($Zn_{1-x}Cu_x$), for NO_2 Adsorption

Zi Wang, Alena M. Sheveleva, Jiangnan Li, Zhengyang Zhou, Sergei Sapchenko, George Whitehead, Mark R. Warren, David Collison, Junliang Sun, Martin Schröder, Eric J. L. McInnes*, Sihai Yang* and Floriana Tuna**

Supporting Information

Analysis of a Cu-Doped Metal Organic Framework, MFM-520($Zn_{1-x}Cu_x$), for NO_2 Adsorption

Zi Wang, Alena M. Sheveleva, Jiangnan Li, Zhengyang Zhou, Sergei Sapchenko, George Whitehead, Mark R. Warren, David Collison, Junliang Sun, Martin Schröder*, Eric J. L. McInnes*, Sihai Yang* and Floriana Tuna*

Dr. Z. Wang, Dr. A. M. Sheveleva, Dr. J. Li, Dr. S. Sapchenko, Dr. G. Whitehead, Prof. D. Collison, Prof. M. Schröder, Prof. E. J. L. McInnes, Prof. S. Yang, Dr. F. Tuna
Department of Chemistry
University of Manchester
Manchester, M13 9PL, UK
E-mail: M.Schroder@manchester.ac.uk, eric.mcinnes@manchester.ac.uk, sihai.yang@manchester.ac.uk, Floriana.tuna@manchester.ac.uk

Dr. Z. Wang, Dr. A. M. Sheveleva, Prof. D. Collison, Prof. E. J. L. McInnes, Dr. F. Tuna
Photon Science Institute
University of Manchester
Manchester, M13 9PL, UK

Dr. Z. Zhou,
State Key Laboratory of High Performance Ceramics and Superfine Microstructure
Shanghai Institute of Ceramics
Chinese Academy of Sciences
Shanghai, 200050, China

Dr. M. R. Warren
Diamond Light Source
Harwell Science Campus
Oxfordshire, OX11 0DE, UK

Prof. J. Sun, Prof. S. Yang
College of Chemistry and Molecular Engineering
Beijing National Laboratory for Molecular Sciences
Peking University
Beijing, 100871, China
E-mail: Sihai.Yang@pku.edu.cn

Index of Supplementary Information

S1 Experimental procedures ... 3
S2 Characterisation Results ... 4
S2.1 Powder X-Ray Diffraction ... 4
S2.2 Infrared Spectroscopy Analysis ... 5
S2.3 Solid state electronic absorption spectra ... 6
S2.4 Thermogravimetric Analysis ... 7
S2.5 Elemental Analysis ... 8
S2.6 Gas Sorption Data ... 9
S2.7 Scanning Electron Microscopy Imaging (SEM) ... 10
S2.8 Single Crystal Structure Analysis ... 11
S3 Electron Paramagnetic Resonance Spectroscopy ... 19
S3.1 Experimental Techniques ... 19
S3.3 Additional CW EPR Spectra ... 20
S3.4 Additional Pulsed EPR Measurements ... 26
References ... 43

S1 Experimental procedures

General procedure for preparing MFM-520($Zn_{1-x}Cu_x$) compounds:

All reagents were used as received from commercial suppliers without purification. Synthesis of H_4L ligand was carried out using the previously reported method.[1] An aqueous solution of copper chloride with concentration 2 mg mL^{-1} was prepared for the synthesis of mixed metal MOFs. MFM-520($Zn_{1-x}Cu_x$) (x=0.005, 0.01, 0.05) compounds were synthesised using varying amounts of metal salts combined with H_4L.

Thermosynthesis of MFM-520($Zn_{1-x}Cu_x$) (x = 0.005, 0.01, 0.5)

A mixture of H_4L (0.1660 g, 0.50 mmol), $ZnCl_2$ (0.1365 g, 0.995 mmol), $CuCl_2$ solution (0.005 mmol, 0.336 mL of $CuCl_2$ solution, 2 g L^{-1} $CuCl_2$ solution) and 2,6-lutidine (0.22 g, 2.00 mmol) were dissolved in water (30 mL) and sealed in a 45 mL Parr bomb. The bomb was heated in an oven at 130 °C for 6 days. Upon cooling to room temperature, a yellow-green micro-crystalline product MFM-520($Zn_{0.995}Cu_{0.005}$) was separated by filtration, washed with de-ionised water, EtOH, Et_2O and then dried in air (Yield: 71%).

The synthesis of other mixed metal materials was conducted using the same method except using different amounts of $CuCl_2$ as listed below. Similar yields were obtained.

MFM-520($Zn_{0.99}Cu_{0.01}$): H_4L (0.1660 g, 0.50 mmol), $ZnCl_2$ (0.1351 g, 0.99 mmol), 0.672 mL of prepared copper chloride solution (0.01 mmol $CuCl_2$) and 2,6-lutidine (0.22 g, 2.00 mmol) was dissolved in water (30 mL).

MFM-520($Zn_{0.95}Cu_{0.05}$): H_4L (0.1660 g, 0.50 mmol), $ZnCl_2$ (0.1297 g, 0.95 mmol), 3.36 mL of prepared copper chloride solution (0.05 mmol $CuCl_2$) and 2,6-lutidine (0.22 g, 2.00 mmol) was dissolved in water (30 mL).

S2 Characterisation Results

S2.1 Powder X-Ray Diffraction

Powder X-ray diffraction (PXRD) measurements were carried out at room temperature on a PANalytical X'Pert PRO diffractometer using CuK_α radiation (λ = 1.5418 Å) at 40 kV, 30 mA, at a scan-speed of 0.034 s^{-1} and a step size of 0.0065 in 2θ. The PXRD pattern for each compound was compared to a simulated PXRD pattern of MFM-520. All PXRD patterns of MFM-520($Zn_{1-x}Cu_x$) (x=0.005, 0.01, 0.05) compounds coincide with the simulated MFM-520 PXRD pattern, indicating identical topology and framework structure.

The sample of NO_2@MFM-520($Zn_{0.995}Cu_{0.005}$) after EPR measurement was tested by PXRD. The PXRD pattern of this sample is consistent with that of MFM-520($Zn_{1-x}Cu_x$) (x=0.005, 0.01, 0.05), indicating that the structure is retained upon exposure to NO_2 for extended periods.

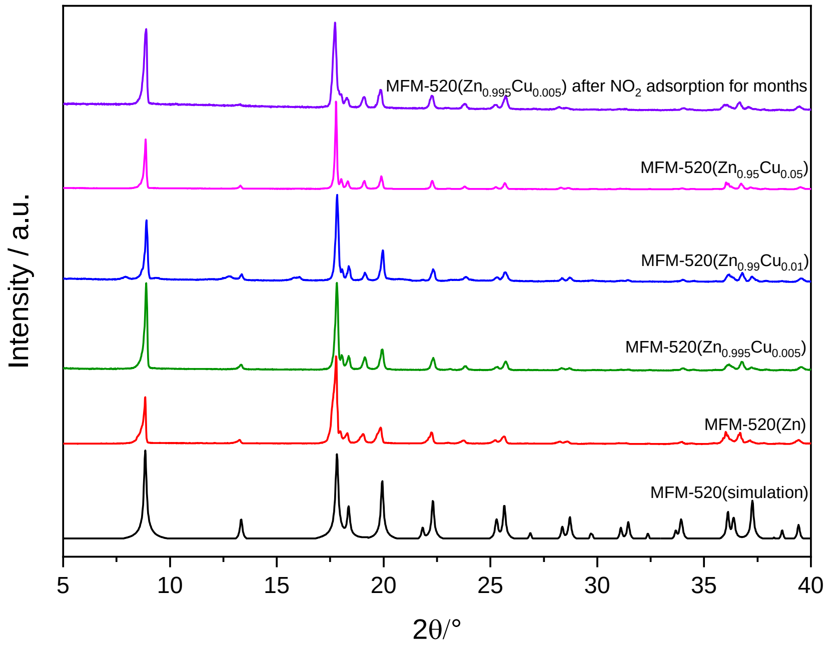

Figure S1. PXRD patterns of MFM-520($Zn_{1-x}Cu_x$) (x=0, 0.005, 0.01, 0.05) and of MFM-520($Zn_{0.995}Cu_{0.005}$) after NO_2 adsorption. The simulated pattern of MFM-520(Zn) is shown for comparison.

S2.2 Infrared Spectroscopy Analysis

Attenuated Total-Reflection Fourier Transform Infrared (ATR-FTIR) spectra were measured on a Nicolet iS5 spectrometer to detect the vibrational modes in the pure and doped materials. No changes were found in the IR spectra of this series, confirming that the vibrational features are retained for all materials in this series.

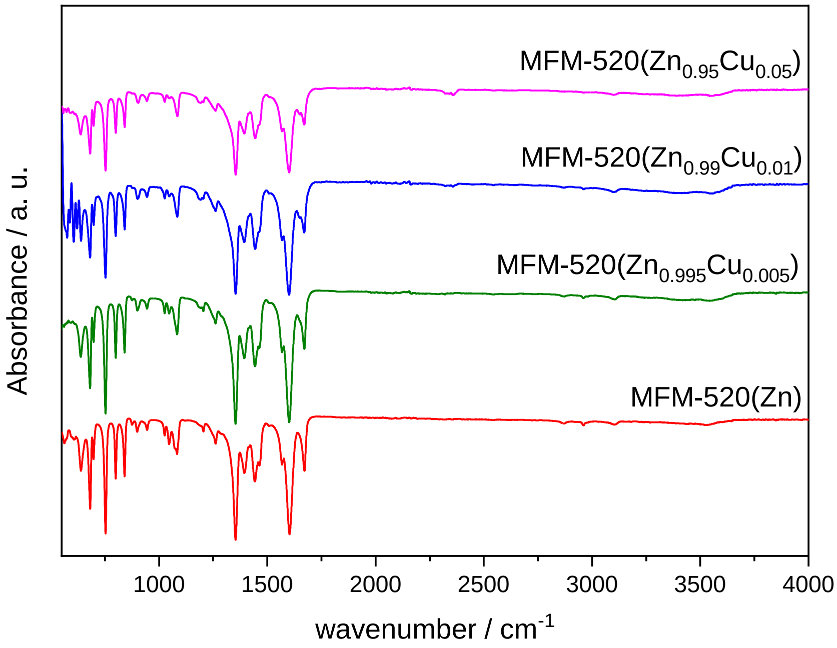

Figure S2. FTIR spectra of MFM-520($Zn_{1-x}Cu_x$) (x=0, 0.005, 0.01, 0.05).

S2.3 Solid state electronic absorption spectra

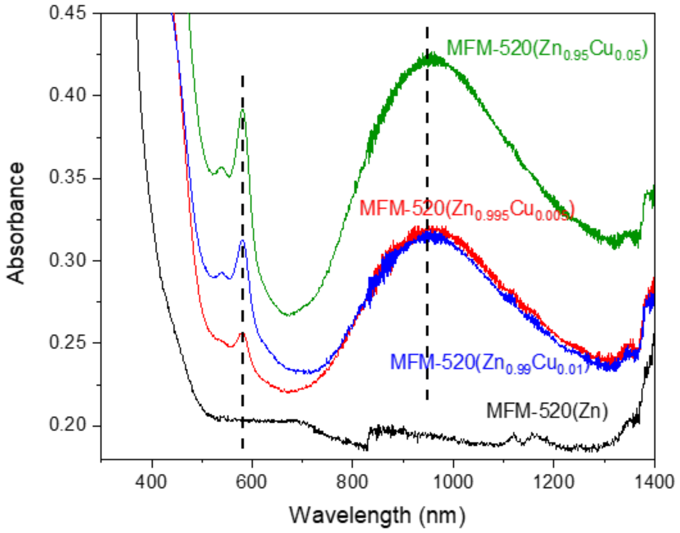

Figure S3. UV/vis/nir-DRS spectra of MFM-520($Zn_{1-x}Cu_x$) (x = 0, 0.005, 0.01, 0.05). Black dotted line: absorption peaks due to *d-d* transitions of Cu^{2+}.

S2.4 Thermogravimetric Analysis

Thermogravimetric analyses (TGA) were performed under a flow of air (20 mL min^{-1}) at a rate of heating of 5 °C min^{-1} to 800 °C using a TA SDT-600 thermogravimetric analyser.

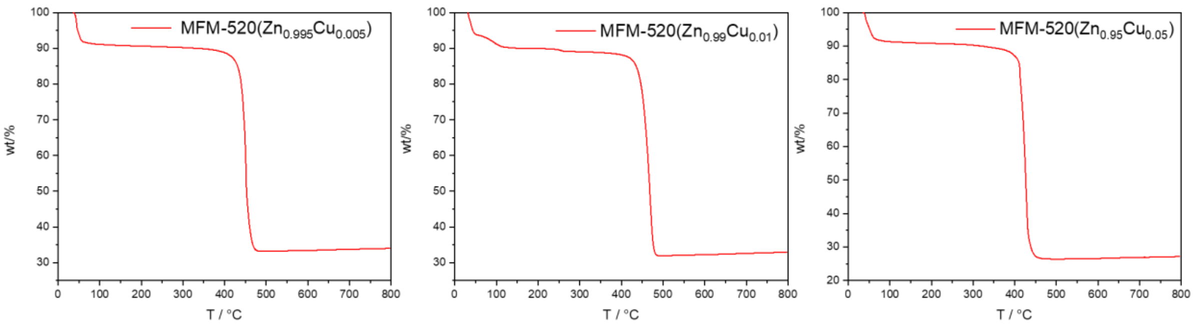

Figure S4. The TGA result of MFM-520($Zn_{1-x}Cu_x$) (x=0.005, 0.01, 0.05).

S2.5 Elemental Analysis

Elemental analysis was performed using a Thermo Scientific iCAP 6000 Series ICP spectrometer and a Thermo Scientific Flash 2000 organic elemental analyser. The amount of copper in MFM-520($Zn_{1-x}Cu_x$) (x = 0.005, 0.01, 0.1) materials was confirmed by ICP-OES method.

Table S1. ICP element analysis results of MFM-520(Cu_xZn_{1-x}).

	x = 0.005		x = 0.01		x = 0.05	
	Expected	Found	Expected	Found	Expected	Found
C(%)	31.7	32.7	31.7	31.3	31.7	32.0
H(%)	2.26	2.34	2.26	2.27	2.26	2.33
N(%)	5.28	5.30	5.28	5.33	5.28	5.20
Zn(%)	24.5	23. 6	24.4	23.3	23.4	23.3
Cu(%)	0.12	< 0.10	0.24	0.20	1.20	1.09

S2.6 Gas Sorption Data

N_2 isotherms were recorded at 77 K on a Micromeritics 3Flex analyser using a liquid nitrogen bath. Gravimetric sorption isotherms of NO_2 were recorded at 298 K on a Hiden Xemis system under ultrahigh vacuum (10^{-10} bar) using a turbo pumping system. The temperature was maintained by a temperature-programmed water bath and furnace,. Ultra-pure research grade (99.999%) NO_2 was purchased from Air Liquide. In a typical gas adsorption experiment, acetone-exchanged MFM-520($Zn_{0.95}Cu_{0.05}$) (50 mg) was loaded into the Hiden Xemis system and activated at 393 K under dynamic high vacuum (10^{-10} bar measured at pump) for 24 h to get fully desolvated material.

Table S2. The BET surface areas of MFM-520(Zn) and MFM-520($Zn_{1-x}Cu_x$) (x = 0.005, 0.001 0.05).

	BET surface area (m^2 g^{-1})
MFM-520(Zn)	313
MFM-520($Zn_{0.995}Cu_{0.005}$)	307
MFM-520($Zn_{0.99}Cu_{0.01}$)	303

MFM-520($Zn_{0.95}Cu_{0.05}$)	309

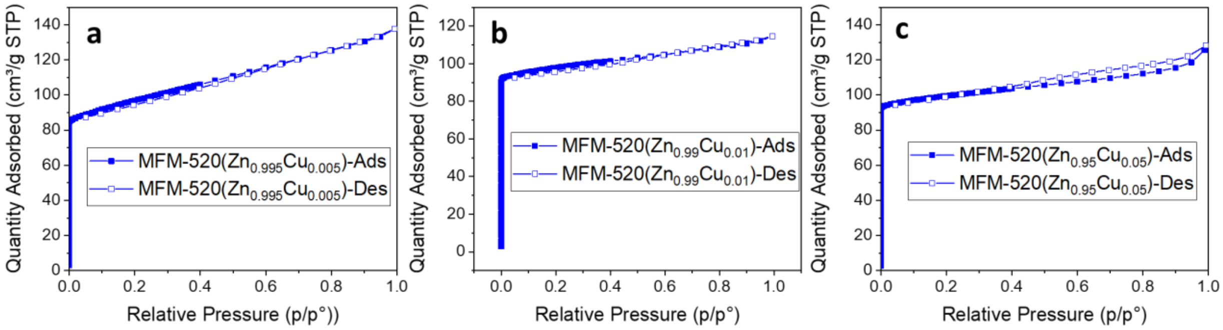

Figure S5. Nitrogen adsorption–desorption BET isotherm of (a) MFM-520($Zn_{0.995}Cu_{0.005}$), (b) MFM-520($Zn_{0.99}Cu_{0.01}$), and (c) MFM-520($Zn_{0.95}Cu_{0.05}$).

S2.7 Scanning Electron Microscopy Imaging (SEM)

All MFM-520($Zn_{1-x}Cu_x$) samples were prepared for SEM by dispersing the material onto a double-sided adhesive conductive carbon tape attached to a flat aluminium sample holder. The samples were imaged at a working distance of 5–6 mm with an accelerating voltage of 10 kV using a low vacuum detector (LVD) on an FEI Nova NanoSEM 230. Carbon coating was used to obtain a higher conductivity.

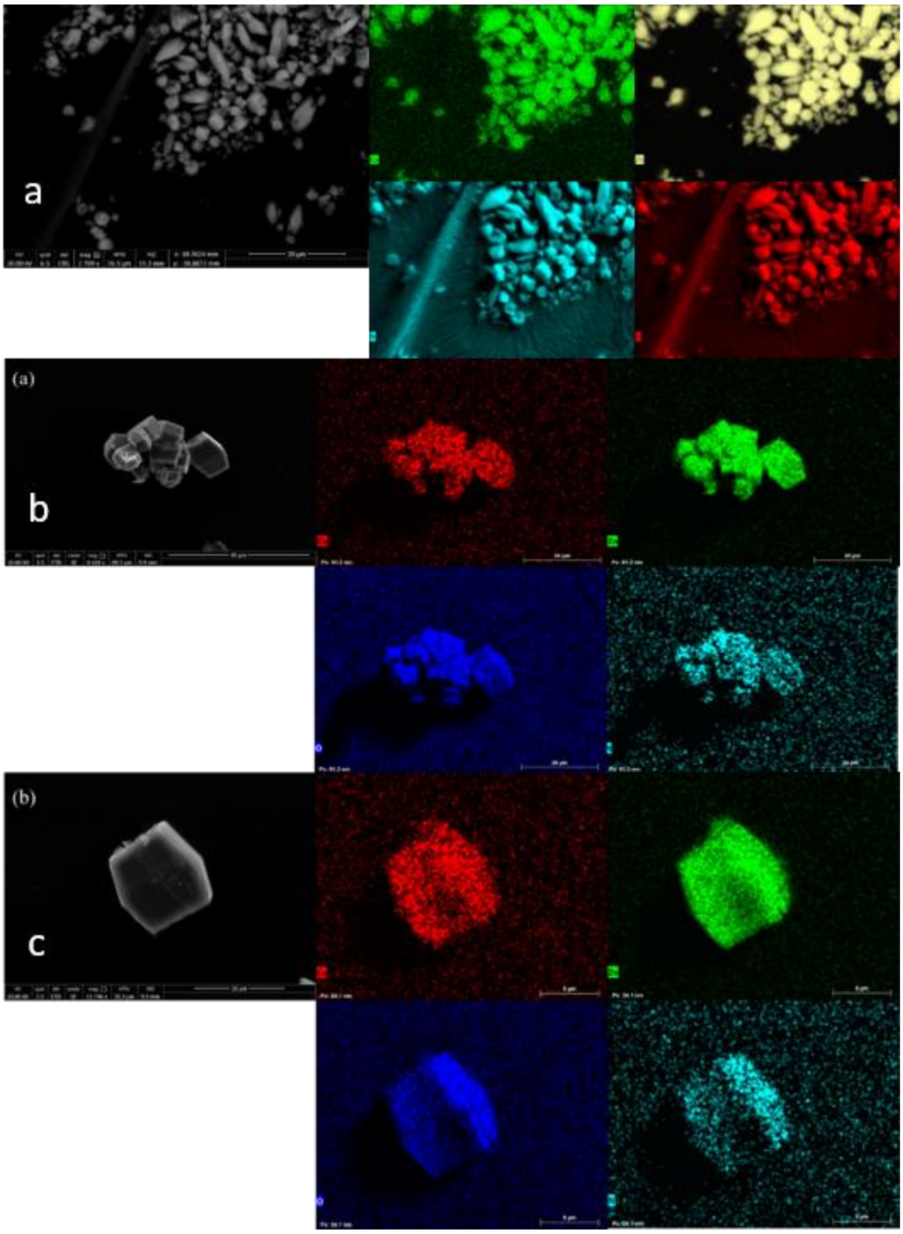

Figure S6. EDS analysis MFM-520($Zn_{1-x}Cu_x$). SEM image (top left) and EDS mapping of the same sample area, demonstrating the presence of elements from ligand. (**a**) $x = 0.005$, scale bar, 20 µm. (**b**) $x = 0.01$, scale bar, 40 µm. (**c**) $x = 0.05$, scale bar, 10 µm (**a**: Cu: green; Zn: yellow; N: blue; O: red. **b and c**: O: blue; N: light blue; Cu: red; Zn: green).

S2.8 Single Crystal Structure Analysis

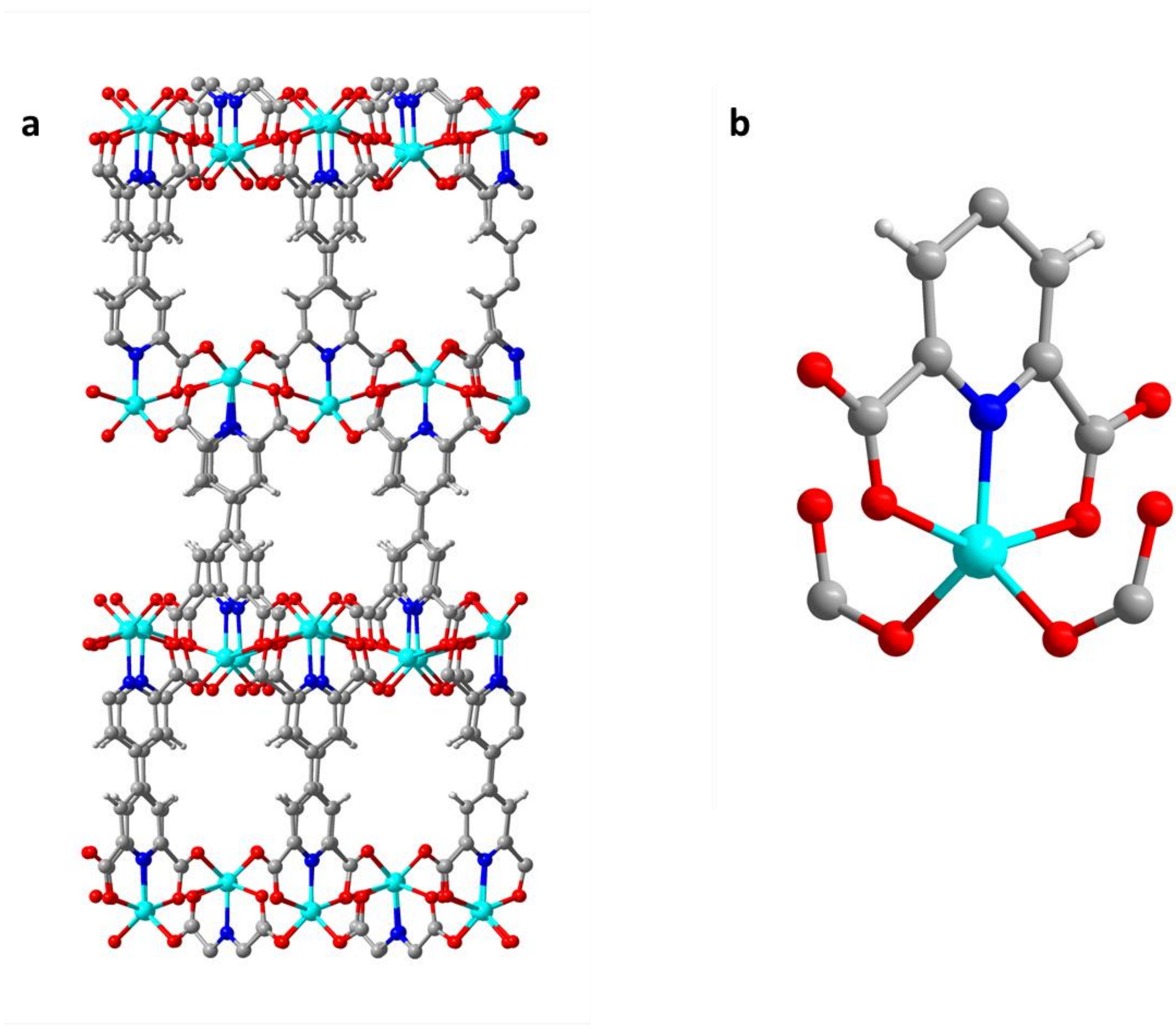

Figure S7. View of (**a**) framework structure of MFM-520($Zn_{0.95}Cu_{0.05}$) and (**b**) the detailed coordination environment of Zn(II)/Cu(II) centres. Zn/Cu: light blue; C: grey; N: blue; O: red; H: light grey.

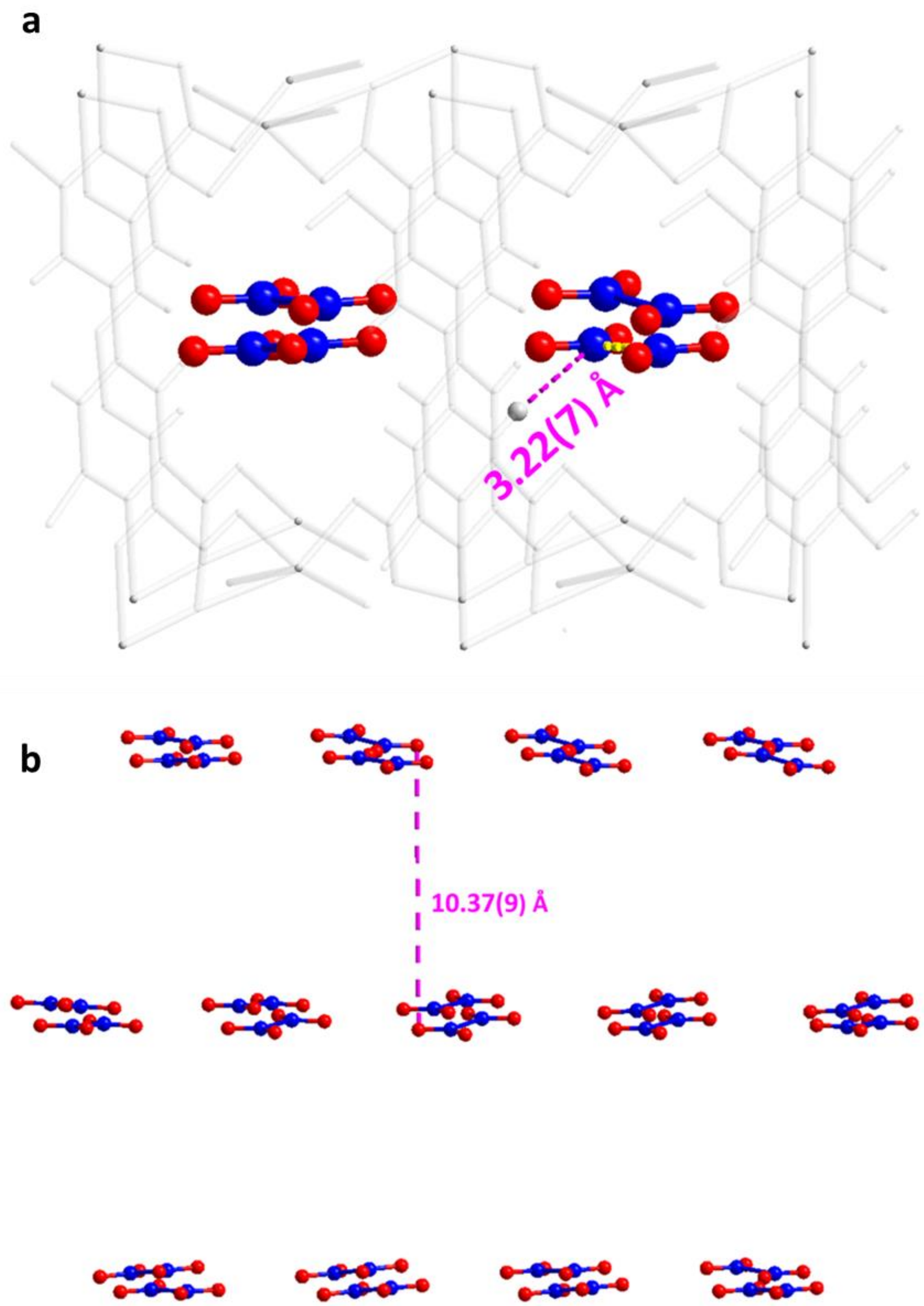

Figure S8 View of (**a**) crystal structure of NO_2-loaded MFM-520($Zn_{0.99}Cu_{0.01}$) with N($\underline{N}O_2$)$\cdots$H(framework) distance, and (**b**) packing of trapped NO_2 molecules within MFM-520($Zn_{0.995}Cu_{0.005}$). The MOF framework has been omitted for clarity.

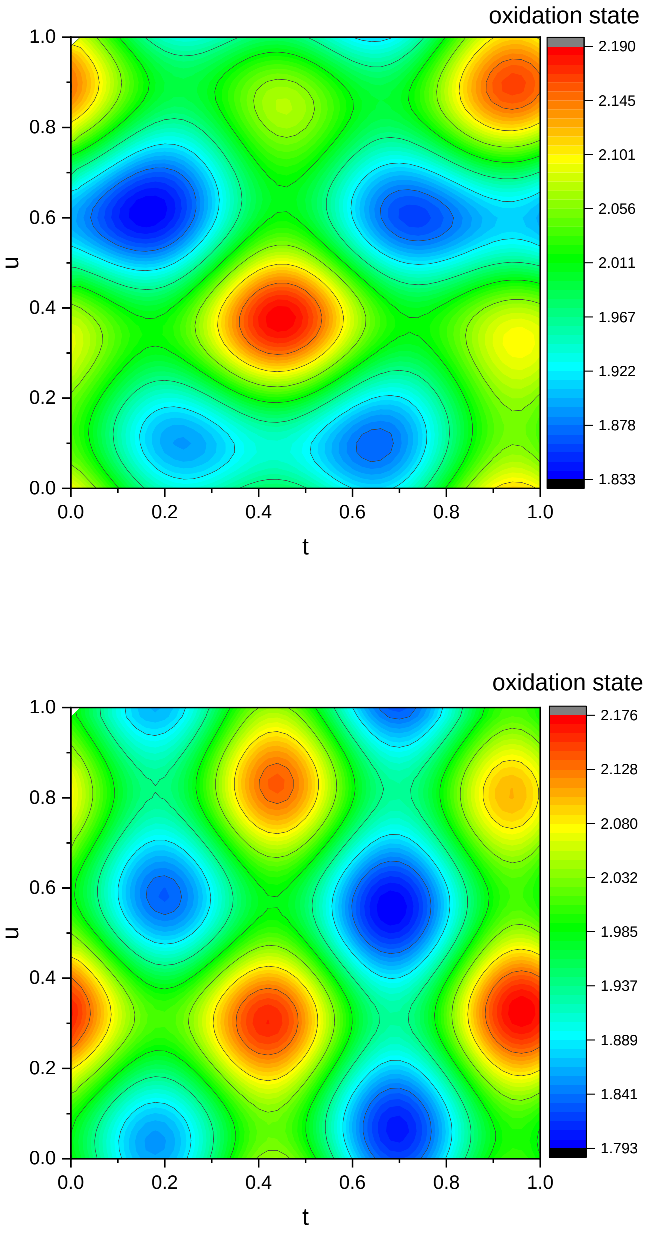

Figure S9. Maps of the bond valence sum (BVS) analyses of the oxidation state of metal centres as a function of the modulation vector u and t in MFM-520($Zn_{0.95}Cu_{0.05}$). Both figures describe the variation of oxidation of metal centres in this material.

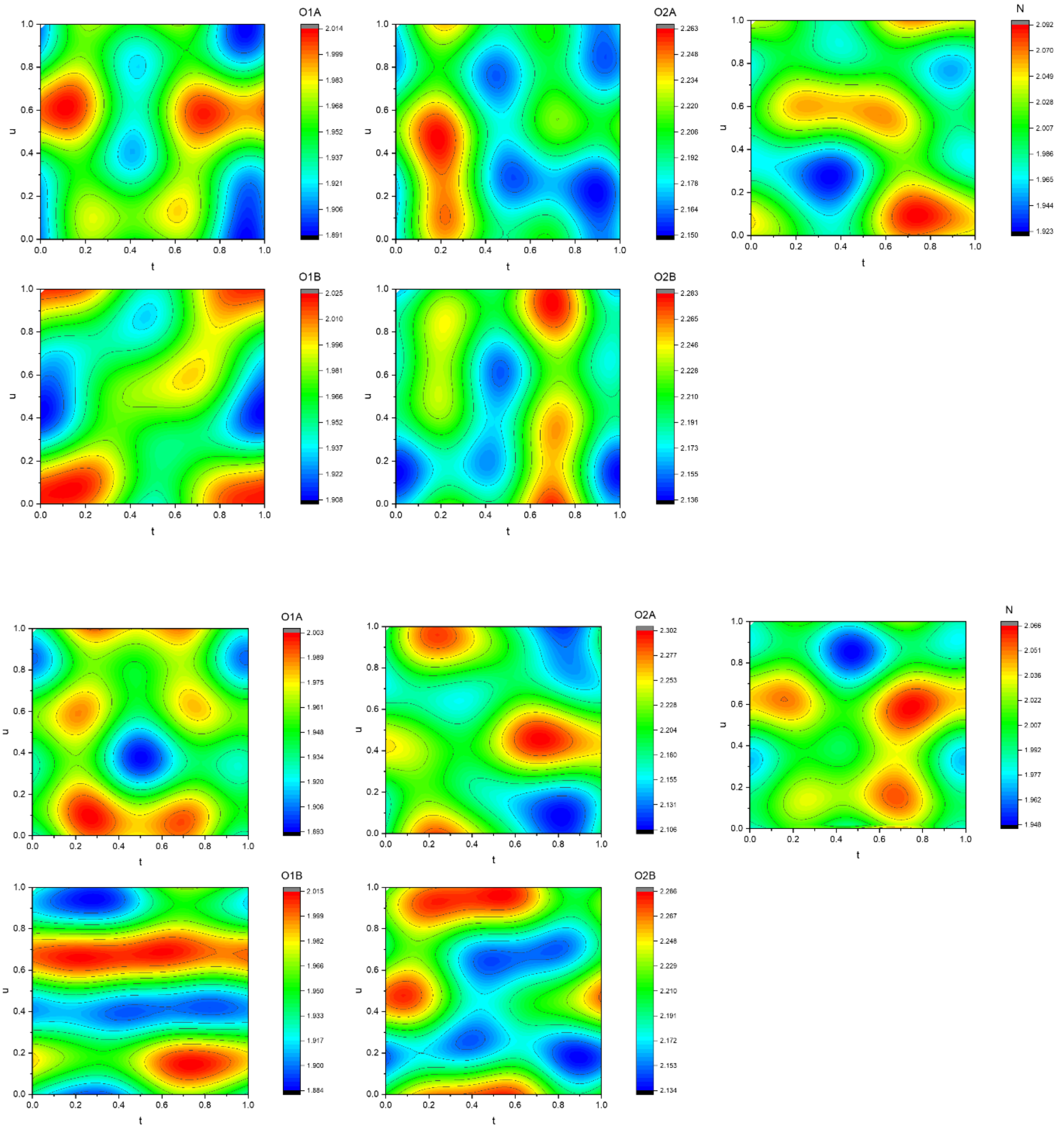

Figure S10. Plot of distances between atoms of [MO_4N] polyhedra as a function of the modulation vector u and t in MFM-520($Zn_{0.95}Cu_{0.05}$). Both sets of figures describe the variations of M-L bonding lengths in this material.

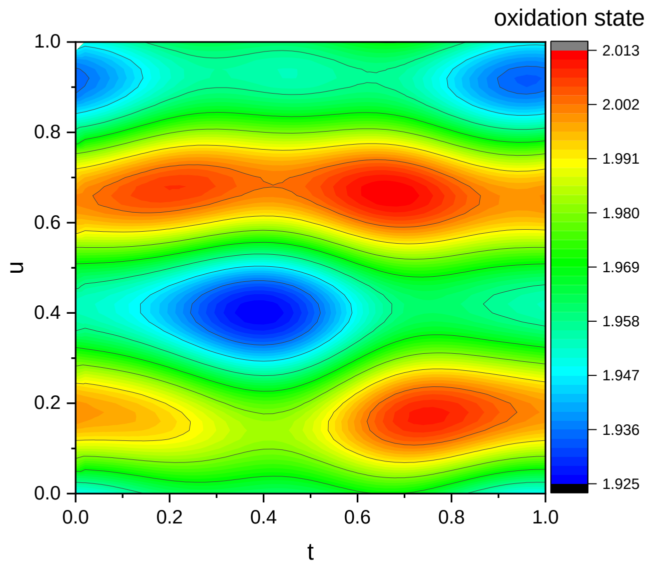

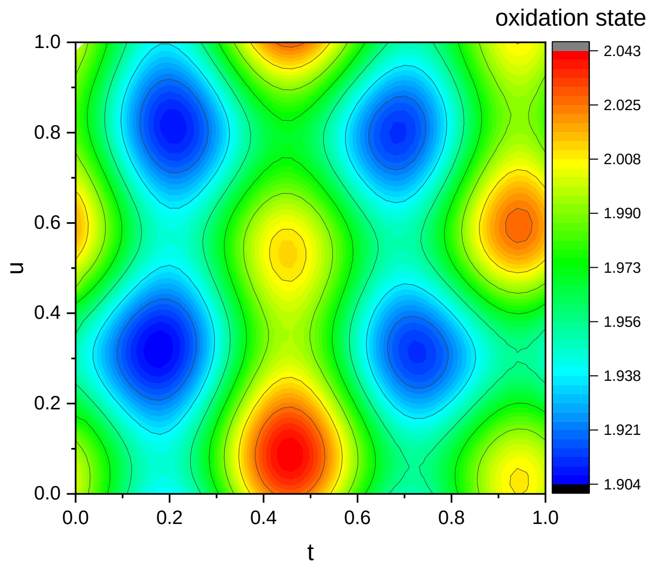

Figure S11. Maps of the bond valence sum (BVS) analyses of the oxidation state of metal centres as a function of the modulation vector u and t in NO_2-loaded MFM-520($Zn_{0.99}Cu_{0.01}$). Both figures describe the variation of oxidation of metal centres in this material.

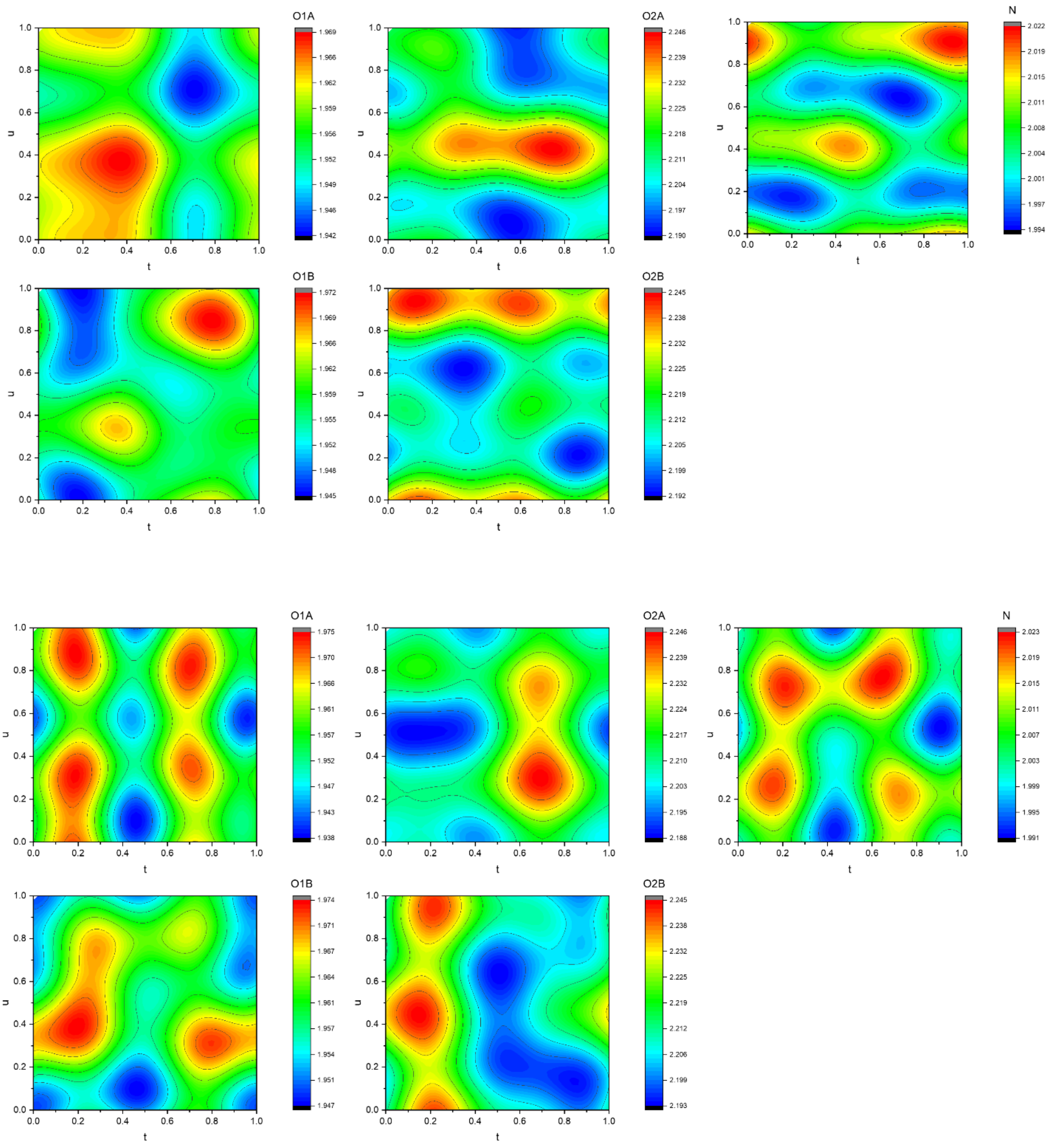

Figure S12. Plot of distances between atoms of [MO_4N] polyhedra as a function of the modulation vector u and t in NO_2-loaded MFM-520($Zn_{0.99}Cu_{0.01}$). Both sets of figures describe the variations of M-L bonding lengths in this material.

Table S3. Summary of crystallographic data

	MFM-520($Zn_{0.95}Cu_{0.05}$)	MFM-520($Zn_{0.99}Cu_{0.01}$)·NO_2
Stoichiometric formula	[$Zn_{1.9}Cu_{0.1}(C_{14}O_8N_2H_4)$]	[$Zn_{1.99}Cu_{0.01}(C_{14}O_8N_2H_4)](NO_2)_{1.614}$

Space group	P-1($\alpha_1\beta_1\gamma_1$)0($\alpha_2\beta_2\gamma_2$)0	P-1($\alpha_1\beta_1\gamma_1$)0($\alpha_2\beta_2\gamma_2$)0
α_1, β_1, γ_1<br>α_2, β_2, γ_2	0.1202(5), 0.1200(5), 0.5005(13)<br>-0.1201(4), 0.1201(5), 0.5007(12)	0.12937(4), 0.12940(6), 0.49987(16)<br>-0.12929(4), 0.12918(6), 0.50004(15)
CCDC	2259399	2259252
a, b, c (Å)<br>α, β, γ (°)	7.0596(3), 7.0616(3), 19.8105(8)<br>89.897(3), 90.050(3), 90.062(3)	7.0707(2), 7.0708(2), 19.8134(5)<br>90.021(2), 90.001(2), 89.996(2)
Temperature (K)	298	150
Environment	vacuum	NO_2
$Theta_{min}$, $Theta_{max}$ (°)	2.07, 27.13	1.46, 27.85
$Reflections_{obs}$ (I > 3σ),<br>$Reflections_{all}$	12335,<br>35483	29406,<br>50635
R_{obs}^{main}, wR_{obs}^{main}<br>R_{all}^{main}, wR_{all}^{main}	0.0767, 0.0736<br>0.1264, 0.0876	0.0925, 0.0797<br>0.1029, 0.0812
$R_{obs}^{\pm(1,\ 0)}$, $wR_{obs}^{\pm(1,\ 0)}$<br>$R_{all}^{\pm(1,\ 0)}$, $wR_{all}^{\pm(1,\ 0)}$	0.0656, 0.0663<br>0.1446, 0.1130	0.0693, 0.0656<br>0.0852, 0.0715
$R_{obs}^{\pm(0,\ 1)}$, $wR_{obs}^{\pm(0,\ 1)}$<br>$R_{all}^{\pm(0,\ 1)}$, $wR_{all}^{\pm(0,\ 1)}$	0.0659, 0.0669<br>0.1402, 0.1105	0.0688, 0.0654<br>0.0843, 0.0710
$R_{obs}^{\pm(2,\ 0)}$, $wR_{obs}^{\pm(2,\ 0)}$<br>$R_{all}^{\pm(2,\ 0)}$, $wR_{all}^{\pm(2,\ 0)}$	0.0720, 0.0799<br>0.2703, 0.2019	0.0695, 0.0685<br>0.1418, 0.1037
$R_{obs}^{\pm(0,\ 2)}$, $wR_{obs}^{\pm(0,2)}$<br>$R_{all}^{\pm(0,\ 2)}$, $wR_{all}^{\pm(0,\ 2)}$	0.0759, 0.0852<br>0.2748, 0.2069	0.0672, 0.0662<br>0.1377, 0.1000
$R_{obs}^{\pm(1,\ 1)}$, $wR_{obs}^{\pm(1,\ 1)}$<br>$R_{all}^{\pm(1,\ 1)}$, $wR_{all}^{\pm(1,\ 1)}$	0.0873, 0.0967<br>0.2598, 0.2149	0.0635, 0.0633<br>0.1246, 0.0988

$R_{obs}^{\pm(1,\ -1)}$, $wR_{obs}^{\pm(1,\ -1)}$<br><br>$R_{all}^{\pm(1,\ -1)}$, $wR_{all}^{\pm(1,\ -1)}$	0.0878, 0.0991<br><br>0.2606, 0.2174	0.0635, 0.0627<br><br>0.1231, 0.0973
R_{obs}, wR_{obs},<br><br>R_{all}, wR_{all}	0.0735, 0.0749<br><br>0.1952, 0.1355	0.0719, 0.0715<br><br>0.1072, 0.0820

S3 Electron Paramagnetic Resonance Spectroscopy

S3.1 Experimental Techniques

Low temperature CW EPR spectra (5 K) of MFM-520($Zn_{1-x}Cu_x$) samples were measured with a Bruker EMX 300 EPR spectrometer equipped with X-band (ca. 9.4 GHz) and Q-band (ca. 35 GHz) resonator and a liquid He cryostat. Field corrections were applied by measuring relevant EPR standards (Bruker Strong Pitch). For accuracy, the tube size and tube position in the cavity were kept constant.

Pulsed EPR measurements of powder samples MFM-520($Zn_{0.995}Cu_{0.005}$) were measured at Q-band (ca. 35 GHz) and X-band (ca. 9.7 GHz) on a Bruker Elexsys E580 spectrometer. Echo-detected field swept (EDFS) spectra were measured at Q-band using the pulse sequence ($\pi/2 - \tau - \pi - \tau$ – echo) with $\pi/2$ and π pulse lengths of 200 and 400 ns respectively. The interpulse delay τ was 200 ns. Electron-nuclear double resonance (ENDOR) measurements used the Davies sequence (π_{inv} − RF −$\pi/2 - \tau - \pi - \tau$ – echo) with microwave inversion and radiofrequency (RF) π-pulse durations of 200 and 1200 ns, respectively[2].

S3.2 EPR sample preparation

Bare MFM-520($Zn_{1-x}Cu_x$) Samples

Acetone-exchanged MFM-520($Zn_{1-x}Cu_x$) (30 mg) was transferred into a J. Young X-band EPR tube (4 and 1.6 mm o.d. for X- and Q-band EPR, respectively). The samples were evacuated at 10^{-5} mbar for 2 h at room temperature, and then activated under dynamic vacuum for 12 hrs at T = 393 K. After the activation process, the EPR tubes were sealed under vacuum using an acetylene flame.

NO_2-loaded MFM-520($Zn_{1-x}Cu_x$) Samples

Acetone-exchanged MFM-520($Zn_{1-x}Cu_x$) (30 mg) sample was evacuated at 10^{-5} mbar for 2 h at room temperature and was then placed under vacuum for 12 h at 393 K to activate the sample. The activated sample was placed into a J. Young EPR quartz tube (4 and 1.6 mm o.d. for X- and Q-band EPR, respectively) equipped with a vacuum valve connected to a NO_2 cylinder incorporating a pressure regulator. The whole system was checked for leakage and the MOF sample was left for 1 h at 1 bar pressure under NO_2. After gas adsorption

was complete the tube was evacuated at 10^{-2} mbar for 1 min, sealed and disconnected from the system at low temperature. The NO_2 gas was condensed into the EPR tube using a cold trap method where the sample volumes containing the MOF material were cooled at a liquid nitrogen temperature $T = 77$ K. The application of the cold trap method during the sealing of the quartz glass tubes ensured that the entire amount of loaded NO_2 was fully trapped within the EPR tubes. Loading of NO_2 was rigorously leak tested and used only within range of a NO_2 detection system with a sensitivity of 0.1 ppm. The *in situ* desorption of NO_2 in a J. Young X-band EPR quartz tube was performed under high vacuum at a pressure of 10^{-5} mbar at $T = 393$ K for 5 h to remove all NO_2 in the sample.

S3.3 Additional CW EPR Spectra

Simulation of the EPR spectra was performed with the EasySpin/MATLAB toolbox, which employs the exact diagonalization of the spin Hamiltonian matrix.[3] The difference between the two Cu isotopes (^{63}Cu and ^{65}Cu) is included in the simulation program, but the effect is not resolved at the line widths observed for this sample.

$$\boldsymbol{H} = \boldsymbol{g}_{||}\boldsymbol{\mu}_{\boldsymbol{B}}\boldsymbol{S}_{\boldsymbol{y}}\boldsymbol{B}_{\boldsymbol{y}} + \boldsymbol{g}_{\perp}\boldsymbol{\mu}_{\boldsymbol{B}}(\boldsymbol{S}_{\boldsymbol{x}}\boldsymbol{B}_{\boldsymbol{x}} + \boldsymbol{S}_{\boldsymbol{z}}\boldsymbol{B}_{\boldsymbol{z}}) + \boldsymbol{A}_{||}\boldsymbol{S}_{\boldsymbol{y}}\boldsymbol{I}_{\boldsymbol{y}} + \boldsymbol{A}_{\perp}(\boldsymbol{S}_{\boldsymbol{x}}\boldsymbol{I}_{\boldsymbol{x}} + \boldsymbol{S}_{\boldsymbol{z}}\boldsymbol{I}_{\boldsymbol{z}}) \quad \text{(Equation S1)}$$

The parameters in the model for a small axially distorted Cu^{2+} system are the g-values $g_{||}$ (= g_y) and $g_{\perp}$ (= g_x ≈ g_z) and the hyperfine coupling constants, $A_{||}$ and $A_{\perp}$. μ_B is the Bohr magneton, B_y, B_x and B_z are the components of the magnetic field, S_y, S_x and S_z are the components of the electronic spin operator and I_y, I_x and I_z are components of the nuclear spin operator. Additional parameters which were used in the fit to obtain the shape of the experimental spectrum are the line shape (which was chosen as Gaussian) and line-width parameters. It is assumed that the g and A matrices are co-linear to simplify the model.

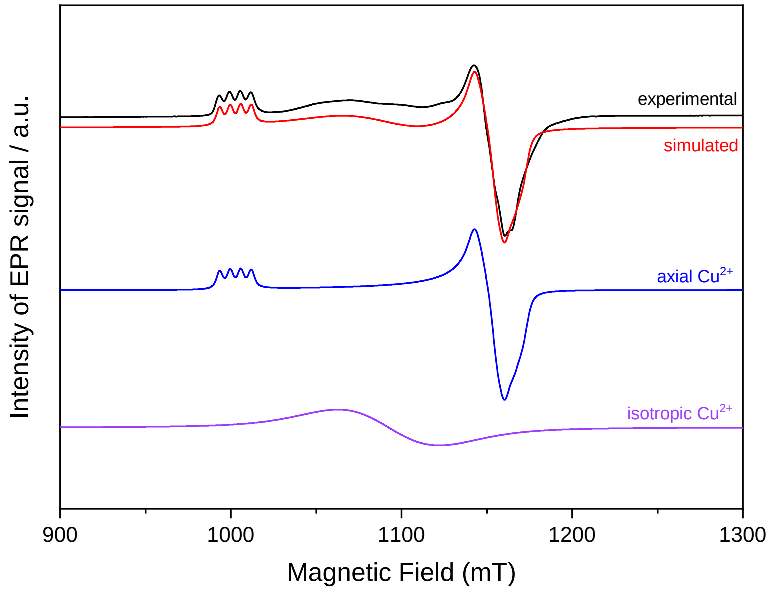

Figure S13. Q-band CW EPR spectrum at 5 K of MFM-520($Zn_{0.95}Cu_{0.05}$) (black), and its simulation (red). The simulation is the sum of the two different species: axial Cu^{2+} (blue) and isotropic Cu^{2+} (purple).

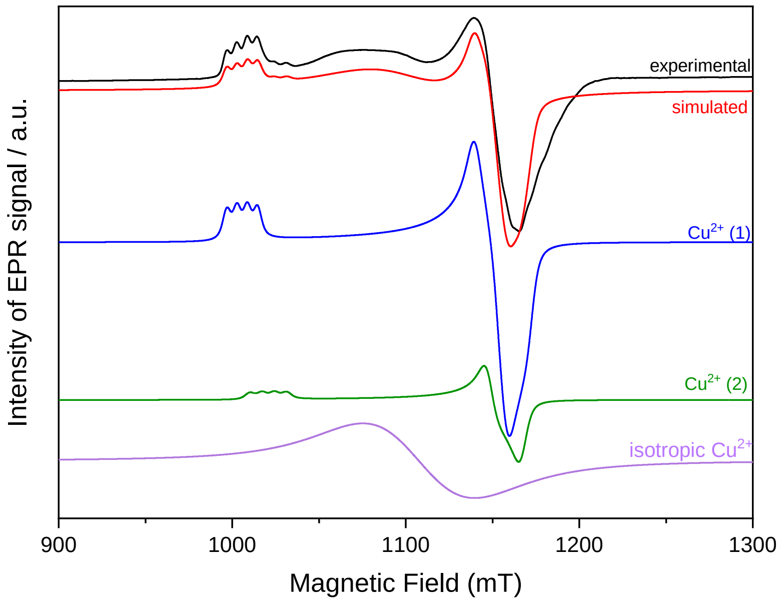

Figure S14. Q-band CW EPR spectrum at 5 K of NO_2-loaded MFM-520($Zn_{0.95}Cu_{0.05}$) (black), and its simulation (red). The simulation is the sum of the three different species: Cu^{2+} (A1) (blue), Cu^{2+} (A2) (dark green), and isotropic Cu^{2+} (purple).

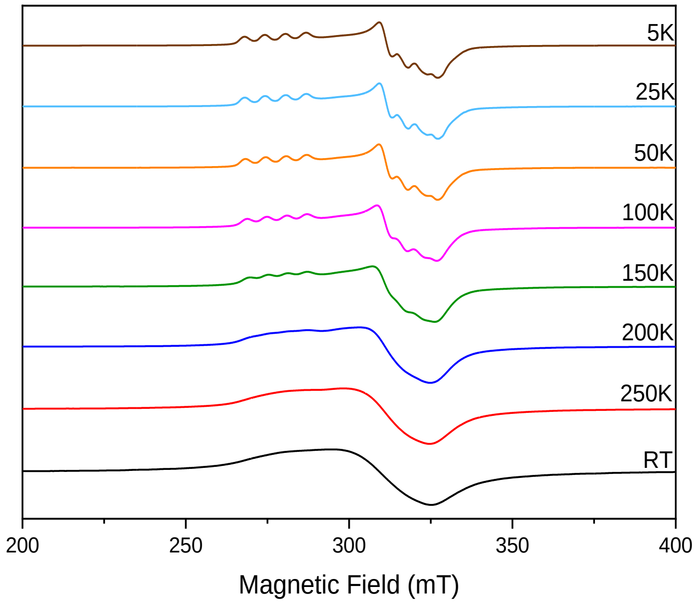

Figure S15. X-band CW EPR spectra of MFM-520($Zn_{0.95}Cu_{0.05}$) from 5 K to the room temperature (298 K).

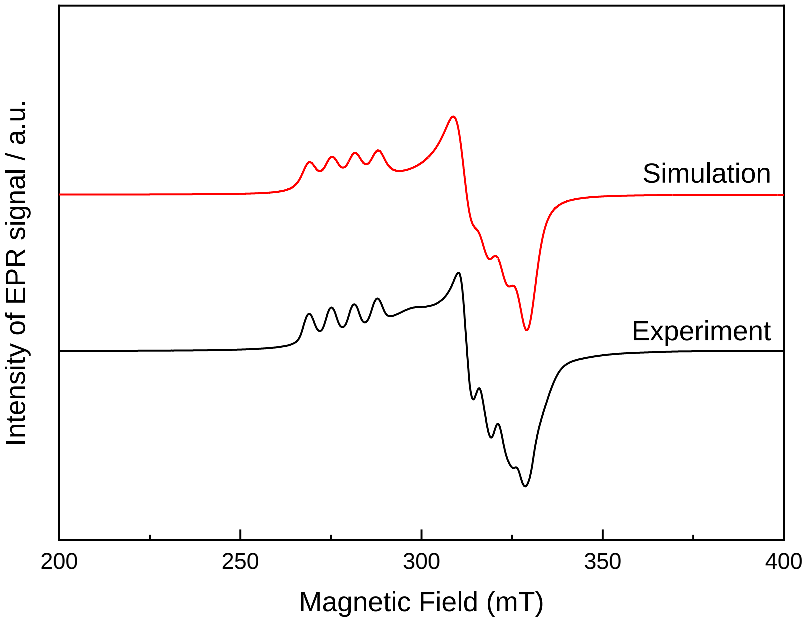

Figure S16. X-band CW EPR spectra at 5 K of NO_2-loaded MFM-520($Zn_{0.95}Cu_{0.05}$) (black), and its simulation (red).

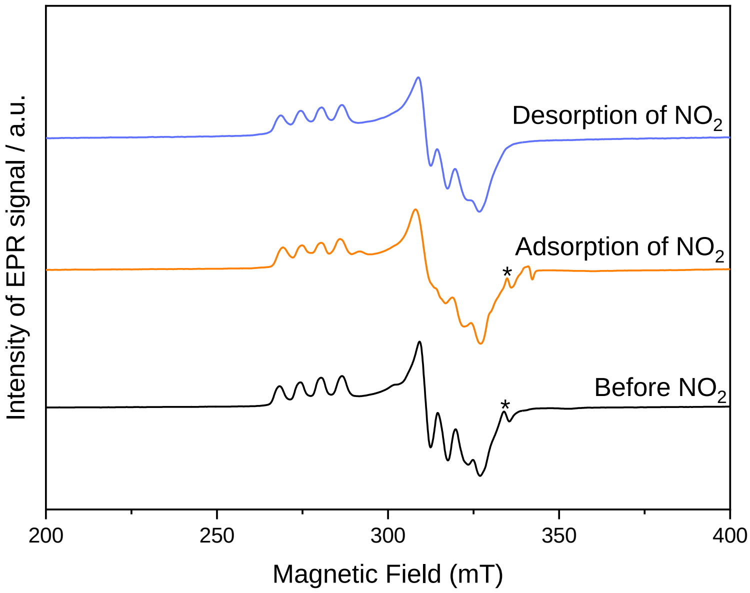

Figure S17. X-band (9.4 GHz) spectra at 5 K of MFM-520($Zn_{0.995}Cu_{0.005}$) before adsorption, after adsorption and after desorption of NO_2. The small peak at $g = 2.0$ (labelled with '*') was identified as coming from the thermocouple which had entered the cavity of the EPR instrument in this particular experiment. This signal is not from the sample.

S3.4 Additional Pulsed EPR Measurements

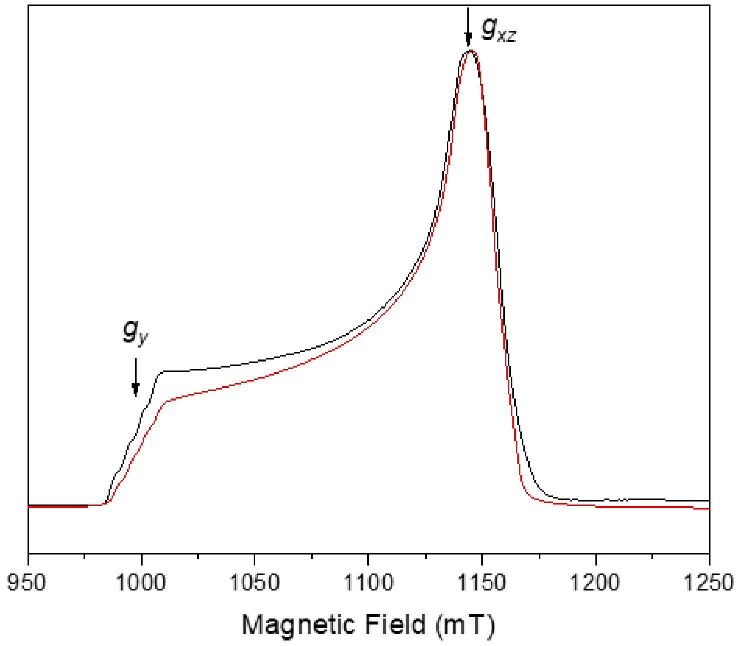

Figure S18. Q-band EDFS spectrum at 5 K for MFM-520($Zn_{0.995}Cu_{0.005}$) (black). The arrows mark the static magnetic field (B_0) positions at which ENDOR was performed. Simulation (red) using the same Hamiltonian parameters as for CW spectrum.

ENDOR spectra show hyperfine interactions between the Cu(II)-based electron spin and ^{1}H nuclear spins of the interior of the MOF. The simulated model was first calculated based on purely dipolar (through space) interactions between Cu and the nearest ^{1}H nuclear. The 3 × 3 dipolar ^{1}H hyperfine interaction matrices ($\mathbf{A}^{\mathrm{dip}}$) were calculated according to:

$$\mathbf{A}^{\mathbf{dip}} = \frac{\mu_0}{4\pi h}\beta_e\beta_n \sum_k \rho_k \frac{3(\mathbf{g}.\mathbf{n}_\mathbf{k})(\tilde{\mathbf{n}}_\mathbf{k}.g_n\mathbf{1})-\mathbf{g}.g_n\mathbf{1}}{r_k^3} \qquad \text{(Equation S2)}$$

where h is Planck's constant (6.63×10^{-34} J s), μ_0 is vacuum permittivity (1.26×10^{-6} T^2 J^{-1} m^3), β_e is Bohr magneton (9.27×10^{-24} J T^{-1}) and β_n is nuclear magneton (5.05×10^{-27} J T^{-1}). The nuclear *g*-values for ^{1}H: $\boldsymbol{g}_\mathbf{H}$ = 5.586. $\boldsymbol{g}$ and $\boldsymbol{g}_\mathbf{n}$

are the electron and nuclear $\boldsymbol{g}$ (3×3) matrixes (g_n is the nuclear g-value, a scalar; **1** is the unit matrix), ρ_k is the electron spin population at atom k ($0 \le \rho_k \le 1$ and dimensionless), n_k is the $n\ldots k$ unit vector expressed in the molecular frame and r_k is the $n\ldots k$ distance (Cu···H).

The vectors n are given by $\begin{pmatrix} \cos\alpha \\ \cos\beta \\ \cos\gamma \end{pmatrix}$ where α, β and γ are the angles of the Cu...H vectors to the defined Cu axes.

There are four nearest H nuclei to the Cu ion and have Cu…H distances shown in Table S4. There are no α-protons at other carbons; the dominant ^{1}H coupling would be from these protons.

Equation S2 is dominated by the copper ion, and for further modelling we assumed ρ_{Cu}= 1. However, we found that the ENDOR calculations with the incorporating dipolar-only parameters with EasySpin software failed to reproduce the experimental data measured both at g_{xz} and g_y orientations because of additional contribution from spin density on the ligand.

Thus, we added in non-zero $\mathbf{A}^{\mathbf{H}^n}$ matrixes from spin polarization contribution to the calculation. Addition of this isotropic hyperfine contribution of this form gives an excellent match to experiment with a_{H1} = 1.85 MHz and a_{H2} = 0.2 MHz. As the isotropic value is positive, it indicates that this isotropic contribution is dominated by the electron transfer from the unpaired electron to the σ system of the ligands, rather than from spin polarization when the unpaired electron delocalized into the ligand π systems (which will give a negative isotropic constant).[4]

Due to the incommensurate modulation of the structure, the geometry of each metal center is similar but not identical, with slightly different M-L distance and angles. As a result, the Cu-H distance and the angles of the Cu...H vectors to the defined Cu axes are changeable. Thus, determine parameters with better agreement, various geometries of metal center with extreme oxidation state or bond length (M1-M4) were selected and extracted from the whole structure and placed into the simulation. The parameters are summarized in Table S4 and calculated spectra are shown Figure S21. The M1 site has the longest O2A/O2B bond length; the M2 site has the highest oxidation state (2.189); the M3 site has the lowest oxidation state (1.793) and the M4 site is closer to the average structure. The calculated spectra from M2 gives the better fitting to experimental spectra.

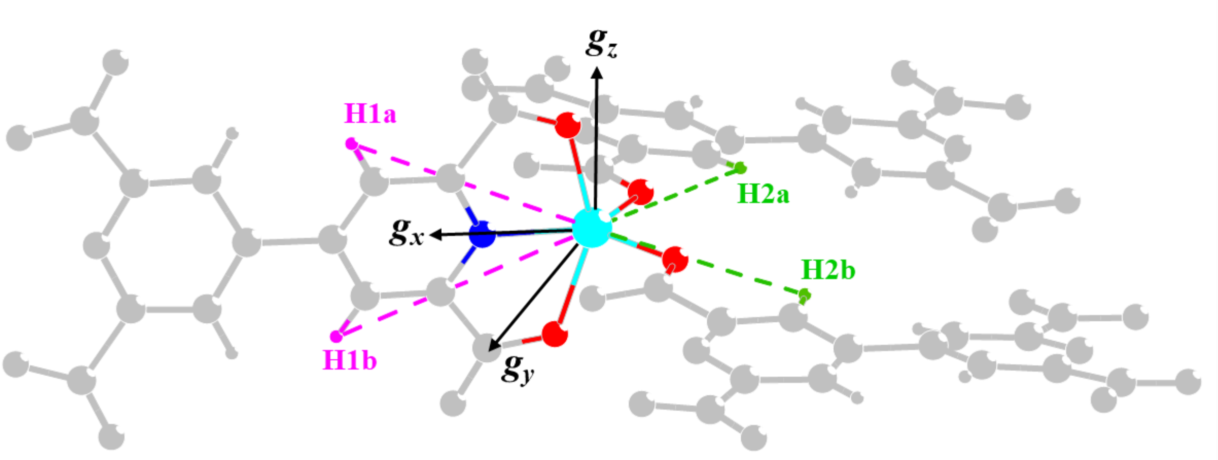

Figure S19. MFM-520($Zn_{1-x}Cu_x$) complex fragment with the definition of *x*, *y*, *z* molecular axes and nearest protons. Cu: light blue, N: blue, O: red, protons with difference Cu-H distance highlighted in different colours.

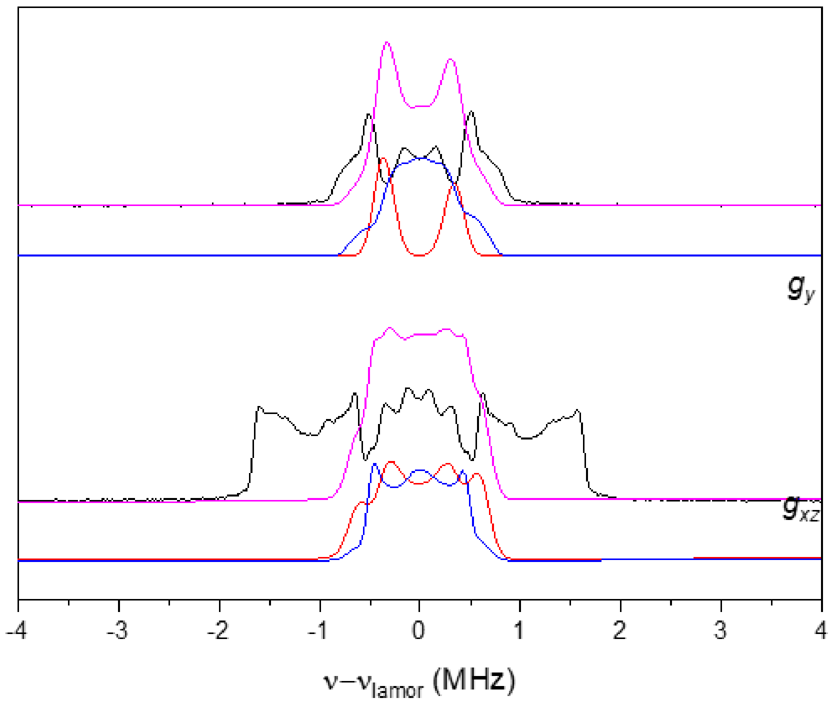

Figure S20. Q-band (33.76 GHz) selective ENDOR experiment spectra (black). Simulation (red and blue: H1 and H2; magenta: sum) was based on a Cu-H point-dipole model.

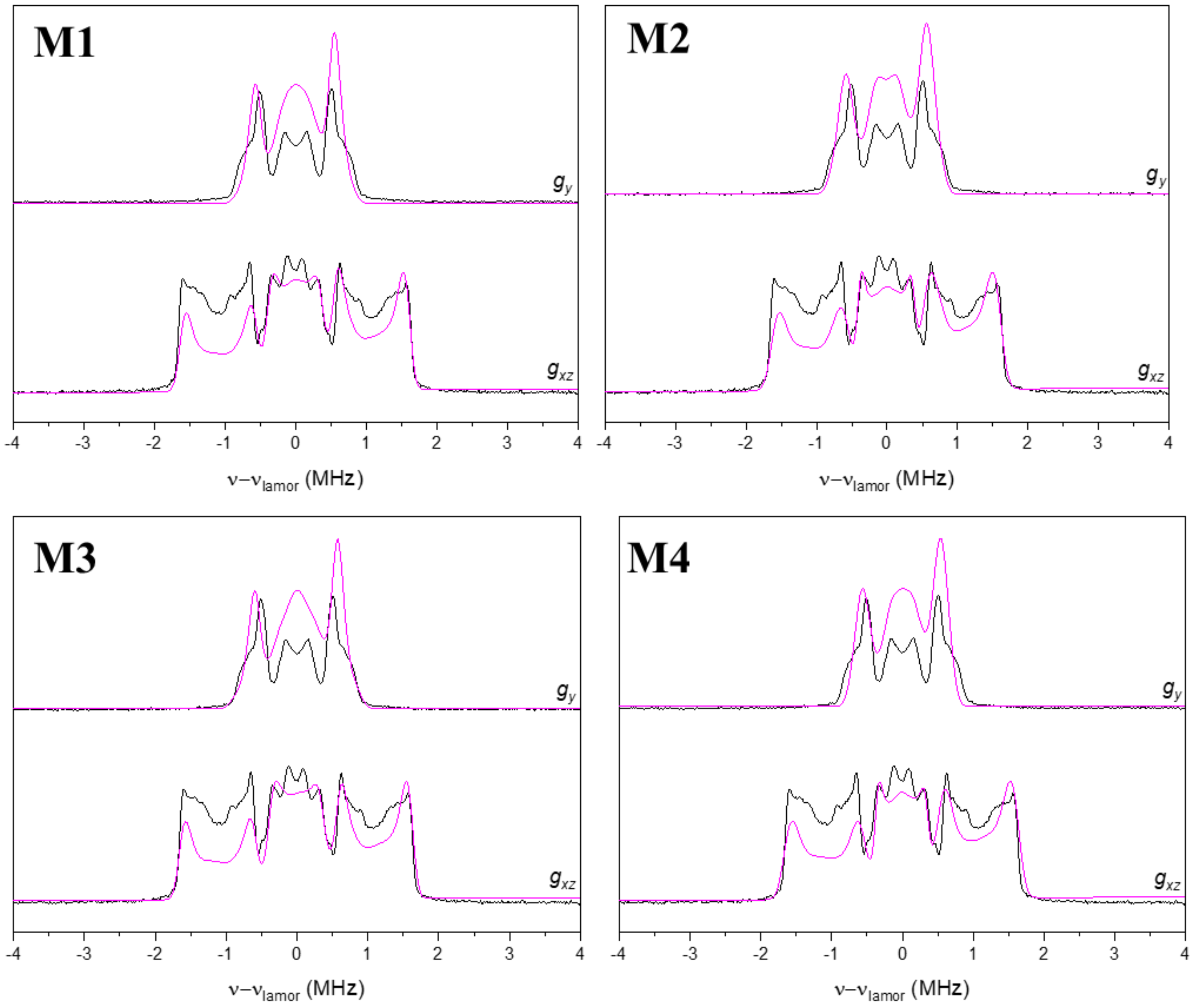

Figure S21. Q-band (33.76 GHz) selective ENDOR experiment spectra (black) at 5 K for MFM-520($Zn_{0.995}Cu_{0.005}$). Simulation (magenta) was based on different geometry of metal centres (M1-M4).

Table S4 Structural parameters from Cu geometry M1-M4, and used for finial calculation of ENDOR spectra in Figure SX.

		α/degree	β/degree	γ/degree	r(Cu-H)/Å
M1<br>u=0.92<br>t=0.36	H1a	21	88.8	69.1	4.915
	H1b	28	87.5	62.2	4.945
	H2a	44.1	46.1	86.5	4.499
	H2b	39.1	52.2	81.4	4.494
M2	H1a	25.6	92.2	64.5	4.942

u=0.88 t=0.94	H1b	22.7	89.7	67.3	4.985
	H2a	43.2	47.9	81.8	4.459
	H2b	39.6	50.5	88.9	4.393
M3 u=0.1 t=0.7	H1a	23.3	90.4	66.7	4.973
	H1b	25.5	89.6	64.5	4.917
	H2a	46.7	43.4	87.5	4.483
	H2b	36.3	54.3	84.4	4.573
M4 u=0.14 t=0.88	H1a	23.9	88.5	66.2	4.910
	H1b	25.2	88.4	64.8	4.860
	H2a	44.1	45.9	89.0	4.660
	H2b	39.1	51.1	87.1	4.587

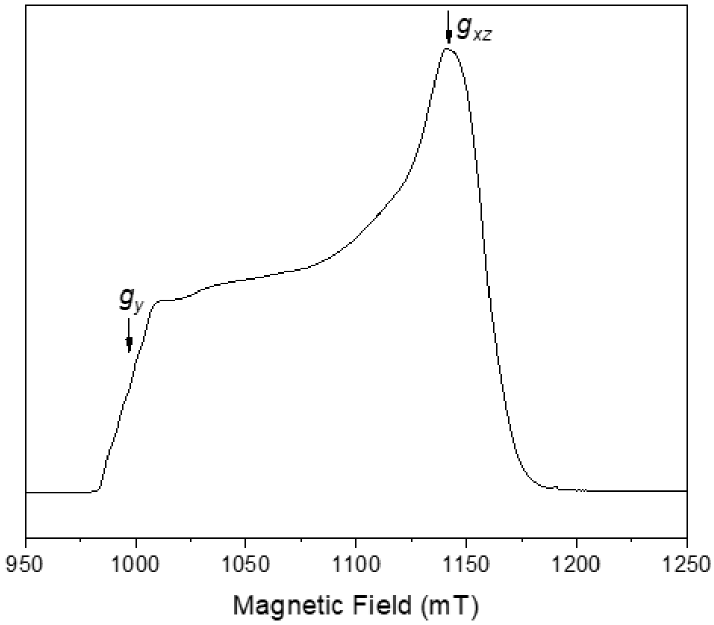

Figure S22. Q-band EDFS spectrum at 5 K for MFM-520($Zn_{0.95}Cu_{0.05}$). The arrows mark the static magnetic field (B_0) positions at which ENDOR was performed.

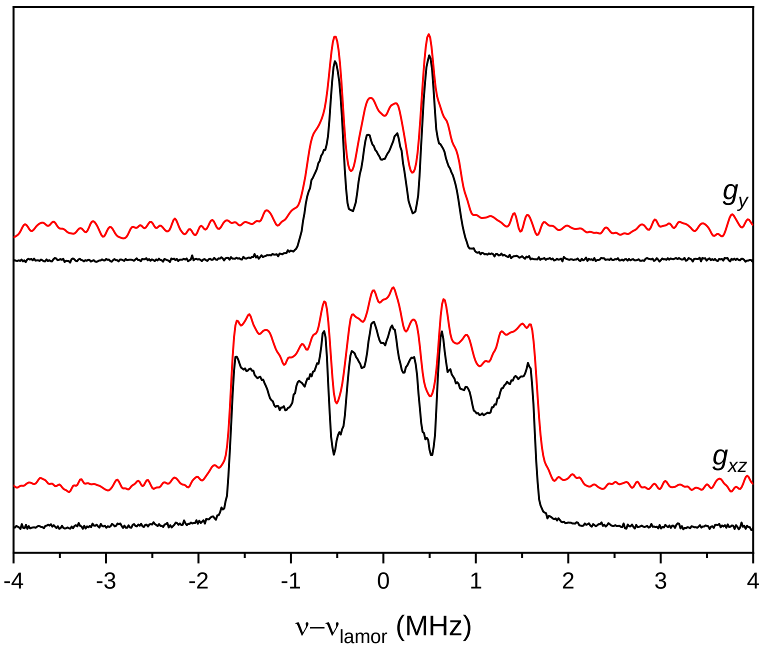

Figure S23. Comparison of ^{1}H ENDOR spectra of MFM-520($Zn_{0.95}Cu_{0.05}$) (red) and MFM-520($Zn_{0.995}Cu_{0.005}$) (black).

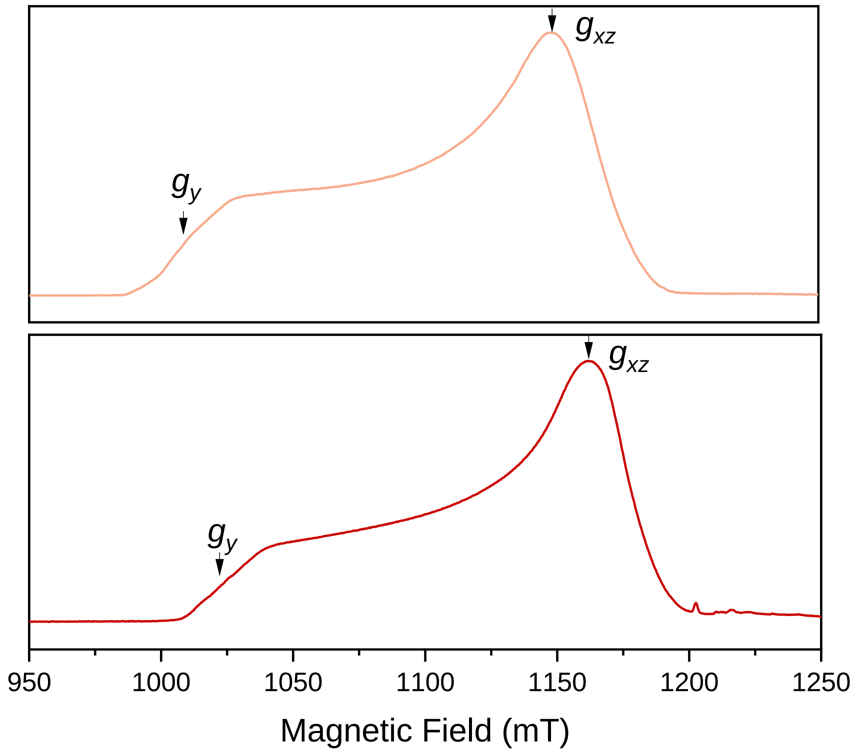

Figure S24. Q-band EDFS spectrum at 5 K for NO_2-loaded MFM-520($Zn_{0.995}Cu_{0.005}$) (upper) and MFM-520($Zn_{0.95}Cu_{0.05}$) (lower).

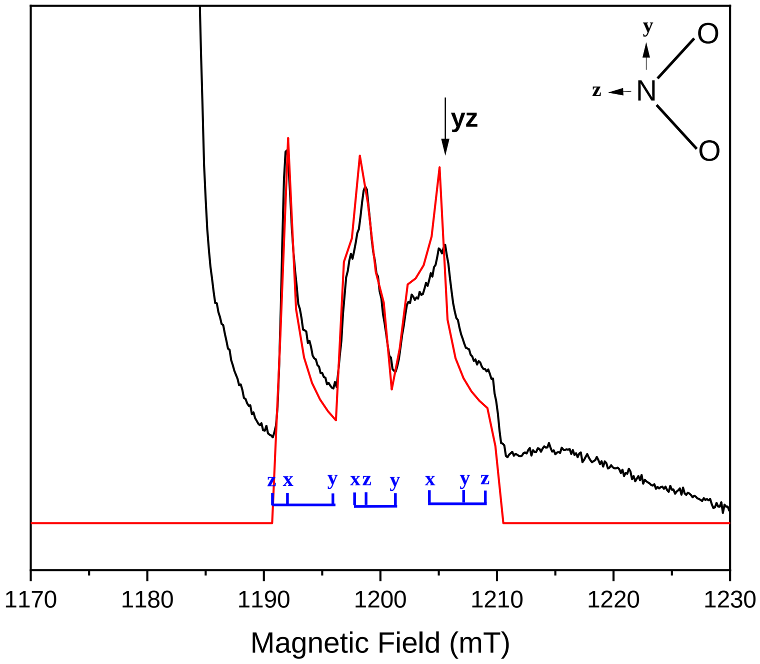

Figure S25. Q-band EDFS spectrum at 5 K for NO_2-loaded MFM-520($Zn_{0.95}Cu_{0.05}$). The magnetic field was centred at the NO_2 signal to maximize the signal intensity.

The ENDOR spectra shows hyperfine interactions between the NO_2-based electron spin and ^1H nuclear spins of the interior of the MOF. The simulated model was calculated based onpurely dipolar (through space) interactions between the NO_2 and the nearest ^1H nuclear. The 3 × 3 dipolar ^1H hyperfine interaction matrices ($\mathbf{A}^{\mathbf{dip}}$) were calculated according to:

$$\mathbf{A}^{\mathbf{dip}} = \frac{\mu_0}{4\pi h}\beta_e\beta_n\sum_k \rho_k \frac{3(\mathbf{g}.\mathbf{n}_\mathbf{k})(\tilde{\mathbf{n}}_\mathbf{k}.\, g_n\mathbf{1}) - \mathbf{g}.\, g_n\mathbf{1}}{r_k^3}$$

here ρ_k is the electron spin population at atom k ($0 \leq \rho_k \leq 1$ and dimensionless), n_k is the $n\ldots k$ unit vector expressed in the molecular frame and r_k is the $n\ldots k$ distance ($O_2\underline{N}\cdots H$). The g-matrix is obtained from the EPR spectra. The NO_2 molecular reference frame was defined as the principal axes of the C_{2v} symmetry NO_2 molecule, with which the molecular g and A_N (the ^{14}N hyperfine matrix of NO_2) axes are coincident:

z axis: parallel to the C_2 axis of NO_2, calculated as the unit vector between N and the midpoint of the O...O atoms;

y axis: parallel to the O...O direction, calculated as the unit vector;

x axis: perpendicular to the plane of the NO_2 molecule, calculated as the cross-product of the y and z unit vectors.

The vectors n are given by $\begin{pmatrix} \cos\alpha \\ \cos\beta \\ \cos\gamma \end{pmatrix}$ where α, β and γ are the angles of the NO_2...H vectors to the molecular NO_2 axes.

Good agreement was achieved with a r (N-H) distance about 3.20 Å. For the observed linewidth, the simulated spectra show low sensitivity to the angles of the NO_2...H vectors to the molecular NO_2 axes.

Table S5. Spin Hamiltonian parameters of MFM-520($Zn_{0.995}Cu_{0.005}$) and NO_2-loaded MFM-520($Zn_{0.995}Cu_{0.005}$) from the simulation of the experimental Q-band CW EPR spectrum and ^{14}N ENDOR spectra.

	MFM-520($Zn_{0.995}Cu_{0.005}$)	NO_2-loaded MFM-520($Zn_{0.995}Cu_{0.005}$)
g_x g_y g_z	2.098, 2.424, 2.112	2.095, 2.385, 2.095
$A_{x,y,z}^{Cu}$ / MHz	120, 210, 140	140, 210, 140
$A_{x,y,z}^{N}$ / MHz	38.0, 30.0, 31.5	38.5, 31.5, 33.2
e^2Qq/h / MHz	-4.4	-4.0
η	0	0

Table S6. ^{14}N(pyridyl) nuclear quadrupole and hyperfine coupling constants (MHz) determined by orientation-selective ENDOR in Cu…pyridyl complexes

	e^2Qq/h	η	$A_{\|\|}^{N}$ [a]	$A_{\perp}^{N}$ [b]	A_{iso}^{N} [c]	p_x [d]	s [e]
MFM-520 ($Zn_{0.995}Cu_{0.005}$)	−4.4	0	38.0	30.8	33.2	4.4	1.8
[Cu(chelidamate)(dmf)] [f]	−2.8	0	51.0	38.9	42.8	7.3	2.4

$[Cu\{(py)_2C(CH_2COMe)(OH)\}_2]^{2+}$ g	−3.0	0.2	45.5	35.0	38.5	6.3	2.1
$[Cu(acac)(bipy)]^{+}$ h	−3.0	0	40	30	33.3	6.0	1.8
$[Cu(py)_4]^{2+}$ i	-2	0	37.4	33.8	35	2.0	

(a) Cu…N_{py} direction, (b) average of other two A^N components, (c) $(A_{||}+2A_{\perp})/3$, (d) N $2p_x$ ($2p_\sigma$) spin density, (e) N 2s spin density, (f) ref 5, (g) ref 4, (h) ref 6, (i) ref 7

Table S7. Principal values of the *g* and hyperfine tensors for NO_2.

**Medium**	g_x	g_y	g_z	A^N_x	A^N_y	A^N_z	**Ref.**
$NaNO_2$ (77 K)	2.0057	1.9910	2.0015	4.94	4.69	6.8	8
$AgNO_2$ (77 K)	2.0090	1.9979	2.0039	5.07	4.85	6.96	8
KNO_3 (77 K)	2.0055	1.9932	1.9996	4.88	5.04	6.30	8
CaX zeolite (77 K)	2.0051	1.9921	2.0017	5.19	4.78	6.76	8
NaX zeolite (77 K)	2.0043	1.9922	2.0015	5.11	4.92	6.75	8
MFM-300 (40 K)	2.0052	1.9915	2.0021	5.13	4.84	6.60	9
MFM-520 ($Zn_{0.995}Cu_{0.005}$) (60 K)	2.0040	1.9894	2.0007	5.13	4.67	6.61	This work
NO_2 gas (293 K)	2.0062	1.9910	2.0019	4.56	4.52	6.60	8

X-band Pulsed EPR Measurements

X-band ENDOR measurements were performed to investigate interactions between N and Cu centers. Echo-detected (ED) spectra were measured at X-band using sequence ($\pi/2$ - τ - π - τ – echo) with $\pi/2$ and π length of microwave (mw) equals 16 ns and 32 ns. The interpulse τ delay was 150 ns. The interactions with ^{14}N ($I = 1$) were probed with ENDOR measurements using the Davies sequence (π_{inv} - RF - $\pi/2$ - τ - π - τ – echo) with microwave inversion and radiofrequency (RF) π-pulse durations of 200 and 1200 ns, respectively.[2] The spectra were obtained at two different values of magnetic field, from g_{xy} (332.4 mT) and g_z (296.0 mT) directions, in order to excite only these portions of the spins which are related to certain molecular orientations.

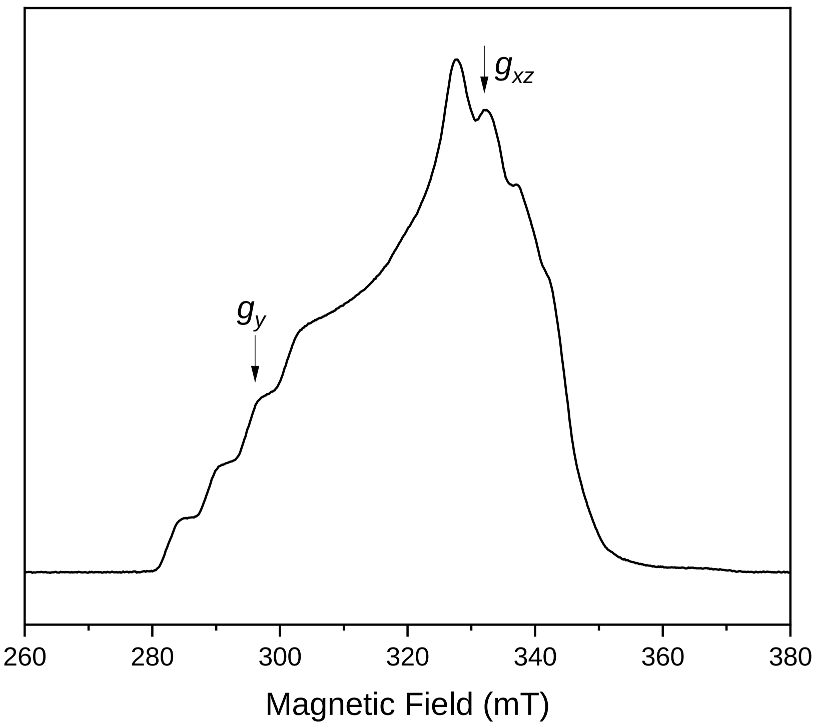

Figure S26. X-band EDFS spectrum of MFM-520($Zn_{0.995}Cu_{0.005}$) at 5 K.

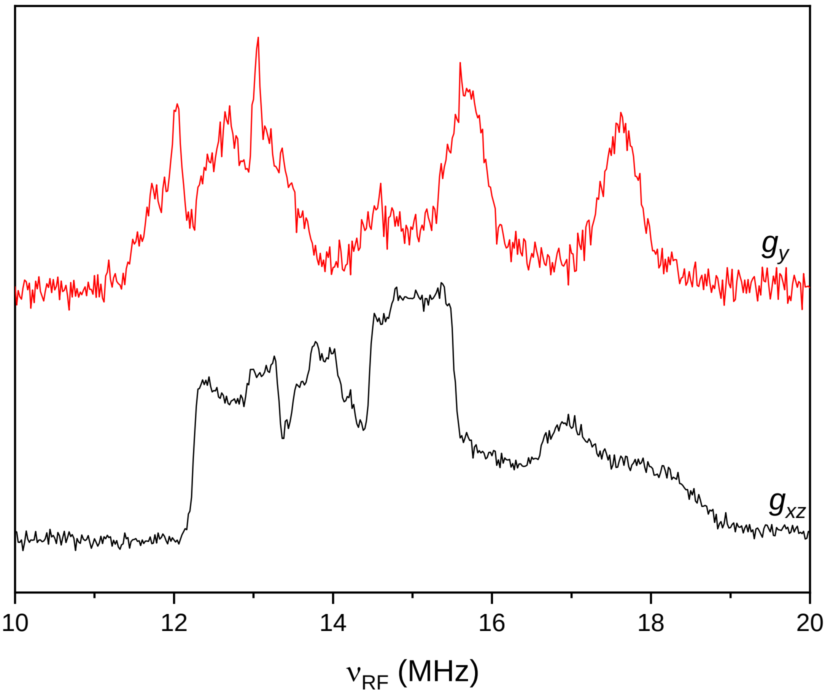

able

Figure S27. X-band (9.79 GHz) ENDOR spectra of MFM-520($Zn_{0.995}Cu_{0.005}$) at 5 K measured at two different static magnetic fields corresponding to xz and y orientations (332.4 mT and 296.0 mT, respectively). Spectra measured with π = 256 ns.

References

[1] X. Lin, A. J. Blake, C. Wilson, X. Z. Xun, N. R. Champness, M. W. George, P. Hubberstey, R. Mokaya, M. Schröder, *J. Am. Chem. Soc.,* **2006,** *128*, 10745-10753.

[2] E. R. Davies, *Phys. Lett. A,* **1974,** *47*, 1-2.

[3] S. Stoll, A. Schweiger, *J. Magn. Reson.,* **2006,** *178*, 42-55.

[4] Z. G. Lada, Y. Sanakis, C. P. Raptopoulou, V. Psycharis, S. P. Perlepes, G. Mitrikas, *Dalton Trans.* **2017**, *46*, 8458-8457.

[5] E. Ramic, R. A. Eichel, K. P. Dinse, A. Titz, B. Schmidt, *J. Phys. Chem. B,* **2006**, *110*, 20655-20663.

[6] A. Folli, N. Ritterskamp, E. Richards, J. A. Platts, D. M. Murphy, *J. Catal.*, **2021**, *394*, 220-227.

[7] H. J. Scholl, J. Hutterman, *J. Phys. Chem.*, **1992**, *96*, 9684-9691.

[8] M. Shiotani, J. H. Freed, *J. Phys. Chem.* **1981**, *85*, 3873–3883.

[9] X. Han, H. G. W. Godfrey, L. Briggs, A. J. Davies, Y. Cheng, L. L. Daemen, A. M. Sheveleva, F. Tuna, E. J. L. McInnes, J. Sun, C. Drathen, M. W. George, A. J. Ramirez-Cuesta, K. M. Thomas, S. Yang, M. Schröder, *Nat. Mater.* **2018**, *17*, 691–696.
